# Raman spectroscopy for nondestructive detection of vaginal tissue alterations in the fibulin-5 murine model of pelvic organ prolapse

**DOI:** 10.1117/1.BIOS.3.3.032106

**Published:** 2026-08-25

**Authors:** Triniti N. Vanoven, Fatemeh Vahidi Zaman, Evelyn T. Pan, Jessica K. Baas, Maria E. Florian-Rodriguez, Kristin S. Miller, Isaac J. Pence

**Affiliations:** a University of Texas Southwestern Medical Center, Department of Biomedical Engineering, Dallas, Texas, United States; b University of Texas at Dallas, Department of Bioengineering, Richardson, Texas, United States; c University of Texas Southwestern Medical Center, Department of Obstetrics and Gynecology, Dallas, Texas, United States; d University of Texas Southwestern Medical Center, Cecil H. and Ida Green Center for Reproductive Biology Sciences, Dallas, Texas, United States; e University of Texas at Dallas, Department of Mechanical Engineering, Richardson, Texas, United States; f University of Texas Southwestern Medical Center, Charles and Jane Pak Center for Mineral Metabolism and Clinical Research, Dallas, Texas, United States; g University of Texas Southwestern Medical Center, Department of Internal Medicine, Dallas, Texas, United States

**Keywords:** raman spectroscopy, pelvic organ prolapse, fibulin-5, planar biaxial mechanics, vagina, mouse model

## Abstract

**Significance:**

Pelvic organ prolapse (POP) is a gynecologic condition where one or more pelvic organs herniate into the vaginal canal that is characterized by suboptimal treatment options, a poorly understood pathophysiology, and a 50% lifetime risk of symptomatic manifestation. The main modality to assess severity is the pelvic organ prolapse quantification (POP-Q) system; however, the POP-Q system, in addition to clinical imaging methods such as MRI and ultrasound, is blind to extracellular matrix (ECM) changes that may relate to loss of tissue compliance.

**Aim:**

We aimed to establish a nondestructive approach to measure optical biomarkers of POP using Raman spectroscopy as well as complementary assays for tissue compliance and remodeling.

**Approach:**

Twenty-four nulliparous fibulin-5 sufficient (wildtype) and deficient (knockout) mice were sacrificed at estrus (20 to 26 weeks old; n=12/genotype) and full thickness vaginal samples (5×5  mm) from the ventral wall were collected. Raman spectra were acquired and averaged from the vaginal epithelium for compositional analysis. Samples were then speckle-coated and tested using planar biaxial protocols to elucidate biomechanical properties. Finally, samples were subjected to histological sectioning and staining for validation. Spectral modeling and statistical analyses were performed, investigating associations between optical and histological composition with biomechanical measures of tissue compliance.

**Results:**

Optical biomarkers of mature, functional elastic fibers significantly decreased in the knockout mice when compared with their wildtype littermates and correlated to histological quantification. In addition, the knockout vagina had direction-dependent alterations in tissue biomechanical response. Nondestructive imaging further identified dysregulated metabolism wherein optical and histological glycogen content significantly decreased when compared with the wildtype controls.

**Conclusions:**

Herein, we have established and verified a workflow for non-destructive compositional analysis with future implications in noninvasive clinical POP evaluation and treatment monitoring. Further, we have established potential optical biomarkers related to dysregulated elastogenesis with POP, such as the $I_{1101}/I_{1654}$ mature, functional elastic fiber to ECM protein ratio.

Statement of Discovery This research combined Raman spectroscopy, histology, and biomechanical testing to elucidate potential optical biomarkers of pelvic organ prolapse (POP) in the fibulin-5 knockout mouse. The synthesis of these techniques represents a critical first step in establishing a translatable noninvasive workflow to monitor POP progression and response to treatment in the clinic.

## 1 Introduction

Pelvic organ prolapse (POP) is a multifactorial condition of impaired pelvic support wherein one or more pelvic organs descend from anatomical position.^1^^–^^4^ Approximately 50% of women will manifest some degree of POP; however, treatment methods remain suboptimal with over 30% of surgical interventions in the United States requiring reoperation after 2 years and noninvasive pessaries limiting everyday activities.^1^^,^^3^^,^^5^^,^^6^ To this end, POP can have a significant impact on everyday life, causing urinary and fecal incontinence, discomfort, embarrassment, body image issues, and sexual dysfunction.^1^^–^^4^^,^^7^ The etiology of POP remains poorly elucidated; however, alterations in critical vaginal extracellular matrix (ECM) components may contribute to impaired tissue function. The interactions between contractile components, such as smooth muscle cells, and noncontractile ECM constituents, including collagen and elastic fibers, dictate vaginal mechanical behavior.^8^^–^^10^ Evidence suggests that elastic fibers permit resilience and compliance, whereas collagen fibers provide tensile strength.^9^^,^^11^^–^^13^ Variations in load-bearing ECM component concentration, arrangement, and/or function may compromise vaginal mechanical and structural integrity; however, the complex relationships between compositional and biomechanical processes with POP are not well defined. Elucidating these biochemical changes and their relationship to tissue functionality may give insight into the vaginal composition-function relationships in the context of POP, thereby providing design criteria for patient assessment and treatment monitoring.

To this end, prior literature implicates dysregulated elastic fiber assembly, specifically decreases in elastin-binding proteins fibulin-5 and lysyl oxidase-like 1, in POP onset and progression.^14^^–^^23^ Indeed, fibulin-5 plays a critical role in the body’s immune response via matrix metalloproteinase (MMP) regulation wherein MMP expression increases in women with severe POP.^24^ Current experimental methods to study these and other alterations in POP’s pathophysiology commonly involve excising tissue, yet biopsies are invasive, time-consuming, and may further compromise vaginal integrity. Mouse models offer controlled, cost-effective, and time-conscious alternatives to human tissues for exploratory studies.^25^ Hence, the fibulin-5 global knockout (*Fbln5*^−/−^) mouse is a validated model of dysregulated elastogenesis that partially recapitulates human POP presentation.^19^^,^^26^ Previous studies have used the *Fbln5*^−/−^ mouse to study composition-function relationships in vasculature,^27^^–^^31^ reproductive organs,^16^^–^^19^^,^^22^^,^^32^^,^^33^ and other biological systems.^34^^–^^36^ Further, $Fbln5^{-/-}$ mice exhibit noticeable signs of malformed elastic fibers as early as 4 to 7 weeks of age; however, nulliparous mice do not manifest signs of POP until later in life.^26^^,^^32^ This allows for a robust and controlled framework to investigate chronological age as a component of POP etiology.^32^

The vagina experiences multiaxial loading in the body. Previous studies indicate alterations in vaginal mechanical properties with POP, resulting in compromised structural integrity.^18^^,^^37^^–^^40^ Further, characterization of vaginal biaxial mechanical behavior is essential for understanding functional changes associated with pelvic organ prolapse and disease progression. The planar biaxial testing protocol controls tissue deformation independently with separate load cells in two orthogonal directions, enabling analysis of tissue anisotropy.^41^^,^^42^ Herein, quantifications of direction-dependent resistances to change in shape and their interplay provide critical insight into ECM functionality.^43^^–^^45^ Despite growing use of biaxial testing in pelvic tissues, the anisotropic mechanical properties in the prolapsed murine vagina are not fully understood.

To elucidate biochemical composition, Raman spectroscopy (RS) is a chemically specific and nondestructive optical technique wherein a sample is excited with near-infrared light to extract a real-time molecular fingerprint indicative of composition. RS is a validated tool for compositional analysis with biomedical applications in the colon,^46^^–^^49^ aorta,^50^^–^^53^ cervix,^54^^–^^59^ and other organs.^60^^,^^61^ Previous studies have coupled RS and biomechanical testing to improve understanding of composition-function relationships in cervical ripening with parturition^57^ and uterine aging.^62^ This evidence suggests that alterations in vaginal composition may correlate with POP-related mechanical dysfunction; however, there exists a significant knowledge gap with respect to vaginal composition-function relationships. Therefore, combining this approach with histological validation and mechanical testing will permit an increased understanding of the vagina with POP.

The objective of this study was to quantify and relate optical composition, passive biomechanics, and histological observations in $Fbln5^{+/+}$ and $Fbln5^{-/-}$ mice. We hypothesize that functional impairments in the $Fbln5^{-/-}$ murine vagina are due to alterations in tissue composition, wherein decreased functional elastic fibers may result in an increased relative collagen concentration.^18^ Further, we anticipate the absence of fibulin-5 will result in inflammation-specific ECM remodeling and dysregulated tissue metabolism.^15^^,^^16^^,^^20^^,^^63^^–^^68^ If successful, this study will increase understanding of POP pathophysiology with potential of RS tool translation for noninvasive ECM monitoring.

## 2 Materials and Methods

### 2.1 Animal Use and Specimen Preparation

All mice used in this study were appropriately housed at 22°C in a 12-h light/dark cycle (0600 to 1800) with access to standard chow and water (Institutional Animal Care & Use Committee at UT Southwestern Medical Center approved; clinical trial number: not applicable). Twenty-four nulliparous C57BL/6J $Fbln5^{+/+}$ (wildtype) and $Fbln5^{-/-}$ (knockout) mice were sacrificed at estrus (20 to 26 weeks old; n=12/genotype) and genotype confirmed via tail snip through a commercial service (Transnetyx, Memphis, Tennessee, USA).

Mouse pelvic organ prolapse quantification (MOPQ) scores were measured following an established protocol.^32^ Briefly, the perineal body length and bulge size were measured in millimeters on both wildtype and knockout mice, whereas the vaginal diameter was recorded if sufficient prolapse was present that resulted in the introitus expanding. Perineal bulge, cervical descent, and anal prolapse were graded on ordinal scales (0 to 4, 0 to 3, and 0 to 2, respectively) as previously described.^32^ Mice with MOPQ scores of 2 or 3 were included in this study as the prolapsed group, whereas knockout mice with scores of 0 or 1 were excluded to establish extremes for deleterious tissue remodeling.^18^ All wildtype mice were confirmed to have a MOPQ score of 0 prior to additional analysis to rule out potential confounding effects.

**Fig. 1:**
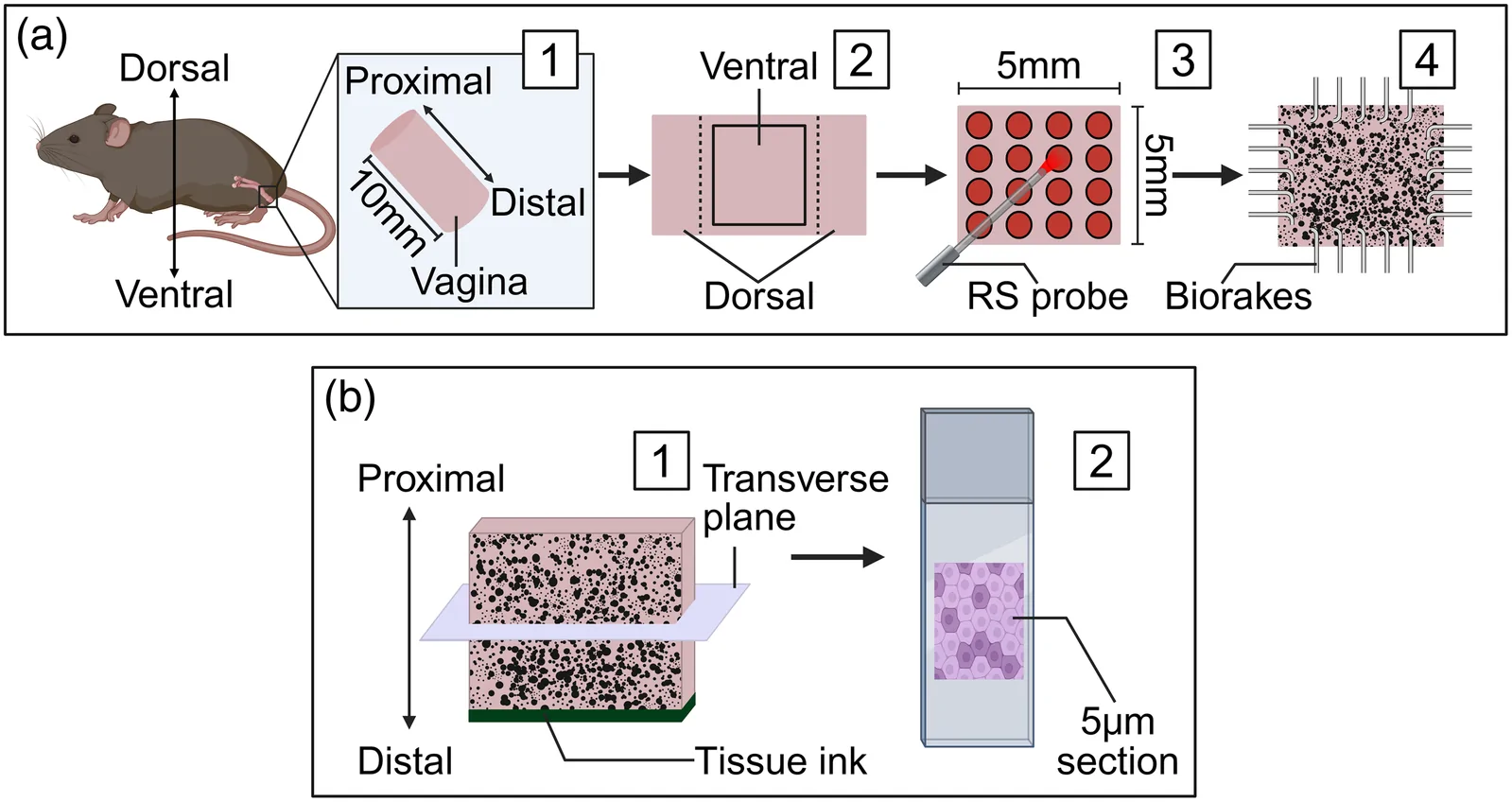
Experimental workflow schematic. Following sacrifice, the reproductive tract was extracted and the vaginal canal isolated (a.1). The vaginal tissue was trimmed laterally to obtain a ∼5×5  mm specimen centered on the medial ventral wall for *ex vivo* analysis (a.2), then probed at sixteen regions of interest using the portable RS system (a.3). The tissue was then speckle-coated and subjected to planar biaxial mechanical testing (a.4). At the conclusion of biomechanical testing, the specimen orientation was marked with tissue ink (b.1) and fixed in 10% neutral buffered formalin for 24 h, then submerged in 70% isopropyl alcohol and stored at 4°C. Finally, specimens were sectioned in the transverse plane in 5  μm serial sections and stained for histological analysis (b.2). Some figure components were made in BioRender.

Following MOPQ assessment, mice were euthanized via cervical dislocation, and the vaginal canal was excised immediately [Fig. 1(a.1)]. An incision was made along the midline of the dorsal vaginal wall to expose the epithelium and a full thickness ∼5×5  mm square was extracted from the ventral wall for *ex vivo* probe-based RS testing [Fig. 1(a.2)]. The unloaded thickness was measured at nine regions of interest in a 3×3 grid using a laser micrometer and averaged (wildtype: 0.205±0.08  mm, knockout: 0.221±0.11  mm, mean ± standard deviation).

### 2.2 Raman Spectroscopy

This study used a high throughput spectrometer (HT3-SPEC-785, EmVision LLC, Loxahatchee, Florida, USA) in combination with a back-illuminated, deep-depleted charge-coupled device cooled to −70°C (PIXIS: 400BR, Teledyne Technologies, Thousand Oaks, California, USA) for optimal noise suppression and quantum efficiency. Further, a superficially biased (∼300 to 600  μm optical penetration) custom Raman probe with seven 300  μm collection fibers surrounding one 200  μm excitation fiber was used (EmVision LLC, Loxahatchee, Florida, USA). The collection geometry included a polished sapphire plano-convex lens paired with a 1-mm-thick magnesium fluoride optical window at the probe tip as previously described.^69^ The 785-nm laser (I0785MM0350 MS-USB, Innovative Photonic Solutions Inc., Plainsboro, New Jersey, USA) excitation was calibrated to 80 mW of power at the probe tip to optimize signal-to-noise ratio while avoiding damage to the tissue, as published in the murine cervix.^57^ On each system boot, the absolute wavenumber axis was calibrated with a neon-argon lamp, and exact Raman shift calculated with spectra from an uncoated acetaminophen tablet and quartz cuvette of naphthalene.^70^ The spectrometer’s spectral resolution of $∼8\;{\mathrm{cm}}^{-1}$, based on the 100  μm slit specification, was confirmed using the full width half maxima of neon-argon spectral emission lines. Spectral response correction was conducted using spectra from a National Institute of Standards and Technology (NIST) standard reference material (SRM2241, NIST, Gaithersburg, Maryland, USA)

Immediately after specimen resection [Figs. 1(a.1)–1(a.2)], a grid of sixteen RS measurements was acquired on the epithelium [ten replicates of 250 ms exposure per grid point; Fig. 1(a.3)]. One knockout specimen was speckle coated prior to RS measurements and subsequently omitted from RS data analyses. Spectral preprocessing included biological fluorescence and background subtraction using a seventh order polynomial fit algorithm in addition to spectral noise smoothing using a Savitzky-Golay filter.^56^^,^^57^^,^^59^^,^^62^^,^^71^^,^^72^

### 2.3 Raman Spectroscopy Data Analysis

Prior to area under the curve normalization, the resulting preprocessed spectra were averaged per grid point to improve the signal to noise ratio. For spectral analysis, all 16 grid points within a specimen were averaged to provide a combined signature per sample. Singular Gaussian distributions were fit to the $1003\;{\mathrm{cm}}^{-1}$ phenylalanine and $1101\;{\mathrm{cm}}^{-1}$ desmosine/isodesmosine peaks to extract position, intensity, and area under the curve (AUC), whereas multiple curves (Gaussian mixture) were fit under the amide I (1590 to $1720\;{\mathrm{cm}}^{-1}$) and amide III bands (1230 to $1300\;{\mathrm{cm}}^{-1}$) for full peak analysis as shown in Table 1 and Fig. S1 in Supplementary Material.^73^

**Table 1:** Characteristic Raman peaks and suggested assignments for peak deconvolution.

Peak (${\mathrm{cm}}^{-1}$)	Tentative assignment	Source(s)
1003	C─C aromatic ring breathing of phenylalanine	55, 73–79
1101	Desmosine and isodesmosine crosslinking amino acids in elastic fibers	79 [Bibr r80] – 81
1246	Amide III mode commonly associated with collagen	56, 59, 75, 78, 82, 83
1300	${\mathrm{CH}}_{2}/{\mathrm{CH}}_{3}$ twisting of lipids	57, 59, 75, 77, 79, 84, 85
1654 (br)	Amide I protein alpha helical secondary structure	55, 59, 74, 75, 79, 86, 87

br, broad.

For supervised advanced biochemical analysis, a non-negative least squares regression (NNLS) was performed to elucidate spectral contributions of pure components on the experimental measurements as previously described.^56^^,^^57^^,^^59^^,^^62^ Unless otherwise specified, all pure components were purchased from MilliporeSigma (Burlington, Massachusetts, USA). Spectra from the pure powdered form of estradiol (human, PHR1353), glycogen (bovine liver, G0885), reverse osmosis water, cholesterol (sheep wool, C8667), elastin (bovine neck ligament, E1625), collagen I (rat tail, C7661), hyaluronic acid (acid form, 935166), actin (rabbit muscle, A2522), progesterone (synthetic, P0130), and phosphatidylcholine (egg yolk, P3556) were collected and processed under the same experimental protocol. A 70/30 ethanol solution was used to clean the probe between measurements; therefore, ethanol was also added to the model to account for potential signal contamination. To satisfy the NNLS assumption of input variable independence, a Spearman correlation analysis was run on the pure spectra and correlations of ρ≥0.65 had one component removed^62^ (Fig. S2 in Supplementary Material). The final model included collagen I (representative of ECM proteins actin, elastin, and collagens I and III), estradiol, progesterone, water, cholesterol, glycogen, ethanol, and lipid (phosphatidylcholine) (Fig. S3 in Supplementary Material). An NNLS regression analysis was conducted to calculate the eight pure components’ contributions on the experimental spectra as previously described.^56^^,^^57^^,^^59^^,^^62^ The outputs from the NNLS model were subsequently normalized to percentage of the total coefficients per spectrum, termed NNLS scores, for ease of comparison.

### 2.4 Biomechanical Testing and Analysis

Following RS measurements, all samples were speckle coated with black India ink for optical deformation tracking (n=12/genotype) [Fig. 1(a.4)]. The tissue was then submerged in Hank’s balanced salt solution and mounted on a biaxial stretching device (BioTester 5000, CellScale, Waterloo, ON, Canada) equipped with two 5N load cells (resolution: ±5  mN). The unloaded grip-to-grip lengths were recorded in both circumferential and longitudinal directions, then a preload of 10 mN was applied to ensure the sample was taut prior to biaxial testing.^45^ The tissue subsequently underwent five rounds of preconditioning to ensure a consistent and repeatable mechanical response followed by a 10-minute equilibration period to ensure consistent and repeatable results^45^ (Fig. S4 in Supplementary Material). Finally, the specimen was subjected to three displacement-controlled protocols of circumferential (θ) to longitudinal (z) ratios 1:1, 1:0.5, and 0.5:1 with a 12% maximum displacement value for five loading and unloading cycles.^45^ A θ:z ratio of 1:1 represents an equibiaxial protocol with 12% maximum displacement in both loading directions, whereas a θ:z ratio of 1:0.5 indicates a maximum displacement of 12% circumferentially and 6% longitudinally. The last loading cycle of all three protocols was used for data analysis wherein anisotropy and direction-dependent tissue properties were evaluated to fit a Fung constitutive model.^45^^,^^88^^,^^89^ Sample deformation was quantified using a MATLAB-based digital image correlation framework as previously described.^90^ Refer to Supplemental Material (Section A) for further details.

### 2.5 Histology

Immediately following biomechanical testing, the tissue was marked with ink on the distal end (closest to the introitus) for orientation [Fig. 1(b.1)], placed in a tissue cassette, and fixed in 10% neutral buffered formalin at room temperature (22°C) for 24 h. At the 24-h mark, the tissue was moved to a 70% isopropanol solution and stored in a 4°C flammable refrigerator until all specimens were collected. The tissues were then submitted to the UT Southwestern Histopathology Core where post-fixation paraffin histology and staining was conducted according to standard procedures.^91^^,^^92^ Briefly, samples were dehydrated, cleared, and embedded in paraffin with 5  μm serial sections cut on the transverse vaginal plane with a rotary blade (RM2255 Microtome, Leica Camera, Wetzlar, Germany)^91^^,^^92^ [Fig. 1(b.2)]. Serial sections were then stained with either hematoxylin and eosin (H&E, blue/purple – nucleus, pink/red – extracellular matrix), modified Hart’s elastin (purple/black – mature, functional elastic fibers), Masson’s Trichrome (MTC, black – nucleus, red – cytoplasm, blue/green – collagen), or an Alcian blue/Periodic acid–Schiff combination stain (AB-PAS, blue – acidic carbohydrates, pink/red – neutral carbohydrates) according to established protocols.^91^^–^^95^

Histological slides were imaged with brightfield illumination using a 20X objective (SLIDEVIEW VS200, Evident, Tokyo, Japan) and analyzed in Fiji.^96^ Images were white balanced and three regions of interest per tissue identified. Following processing, two samples (n=1/genotype) were rendered incompatible with histological analyses and were subsequently omitted. The mature, functional elastic fibers (stained purple/black) were isolated via color thresholding and binarization following an established protocol that was lab-modified for Fiji.^95^ The area fraction of mature, functional elastic fibers was calculated as the number of pixels above the threshold divided by the total number of pixels in the subepithelium and muscularis layers from the Hart’s elastin slides.^11^^,^^18^ Similarly, epithelium and combined subepithelium/muscularis thicknesses were extracted from the aforementioned regions of interest on MTC sections using a lab-modified version of the ThicknessTool ImageJ plugin^97^ (Fig. S5 in Supplementary Material). Finally, AB-PAS-stained sections were deconvolved into red, green, and blue channels using the Colour Deconvolution Fiji plugin and the intensity ratio of the red to blue channel areas was used to represent the ratio of neutral to acidic carbohydrates, respectively,^98^ (Fig. S6 in Supplementary Material).

### 2.6 Statistical Analysis

All statistical analyses were performed in RStudio (R 4.4.2). The grid of 16 RS measurements was averaged per specimen for all statistical comparisons. Data were assessed for normality and homogeneity of variance using Shapiro–Wilk’s and Levene’s tests, respectively. RS features and Fung constitutive modeling parameters were compared between knockout mice with severely prolapsed tissues (KO, MOPQ=2/3) and wildtype mice with nonprolapsed tissues (WT, MOPQ=0) using two-sided Wilcoxon rank-sum tests, except for glycogen NNLS score, histological neutral to acidic carbohydrate ratio, histological elastic fiber area fraction, and $I_{1101}/I_{1654}$ peak ratio, which were modeled as one-sided tests as we hypothesized these parameters would decrease with POP in line with prior literature.^11^^,^^18^^,^^62^^,^^99^ Spearman correlation analyses were conducted between biomechanical parameters, RS features, and histological output to elucidate potential optical biomarkers associated with disease severity.

## 3 Results

### 3.1 Raman Spectroscopy

**Fig. 2:**
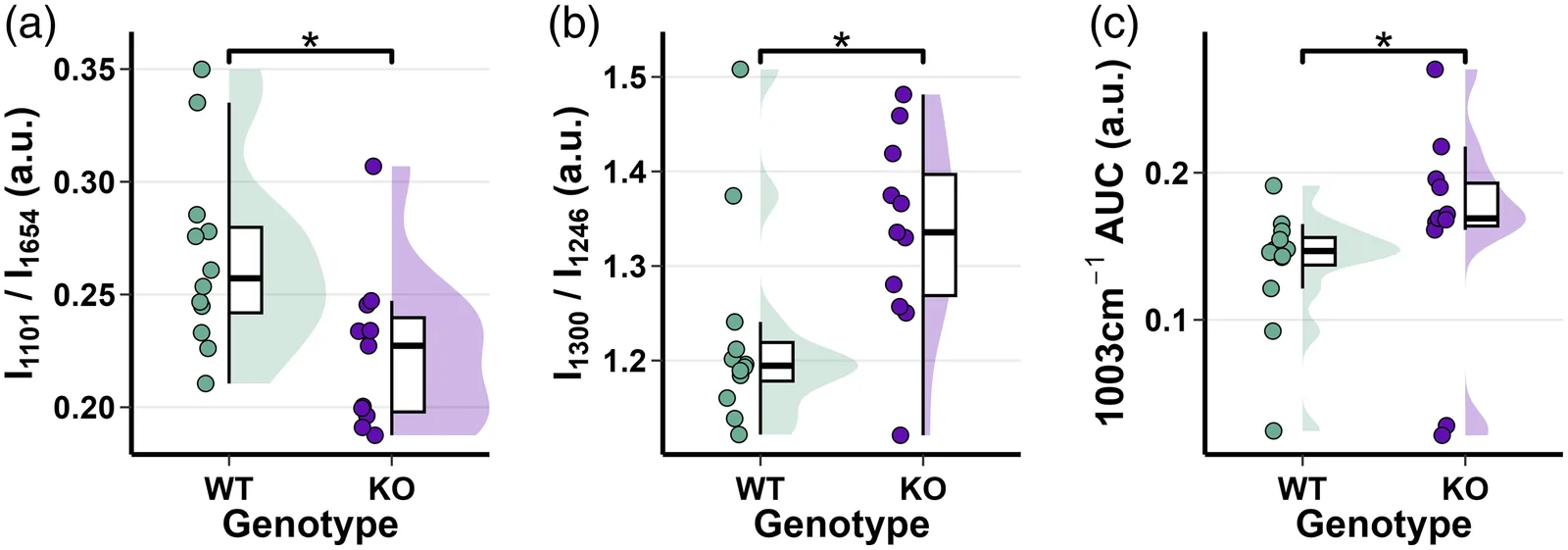
Peak analyses of Raman spectra indicate significant changes in the knockout vagina compared to controls. The $I_{1101}/I_{1654}$ crosslinked elastic fiber to ECM protein peak ratio (a) significantly decreased, whereas the $I_{1300}/I_{1246}$ lipid to protein peak ratio (b) and $1003\;{\mathrm{cm}}^{-1}$ AUC (c) significantly increased in the knockout (KO, MOPQ=2/3, purple, n=11) mice when compared with their wildtype (WT, MOPQ=0, green, n=12) littermates. Tentative peak assignments are found in Table 1. Each excised (5×5  mm) ventral vaginal wall was subjected to 16 RS measurements in grid format [Fig. 1(a.3)], and the results averaged for one data point per mouse. Boxplot data is represented as the mean +/− interquartile range. ***p<0.001, **p<0.01, *p<0.05 denotes statistical significance. One knockout specimen was speckle coated prior to RS measurements, thus omitted from this section of analysis.

Wilcoxon rank-sum tests identified significant differences in peak parameters associated with several ECM components (Fig. 2). In the knockout group, the relative amount of crosslinked elastic fibers to ECM proteins peak ratio $I_{1101}/I_{1654}$ (p=0.015) [Fig. 2(a)] significantly decreased when compared with their wildtype littermates. Conversely, the lipid to protein ratio $I_{1300}/I_{1246}$ (p=0.019) [Fig. 2(b)] and $1003\;{\mathrm{cm}}^{-1}$ AUC (p=0.027) [Fig. 2(c)] significantly increased in the knockout group compared to the wildtype controls.

**Fig. 3:**
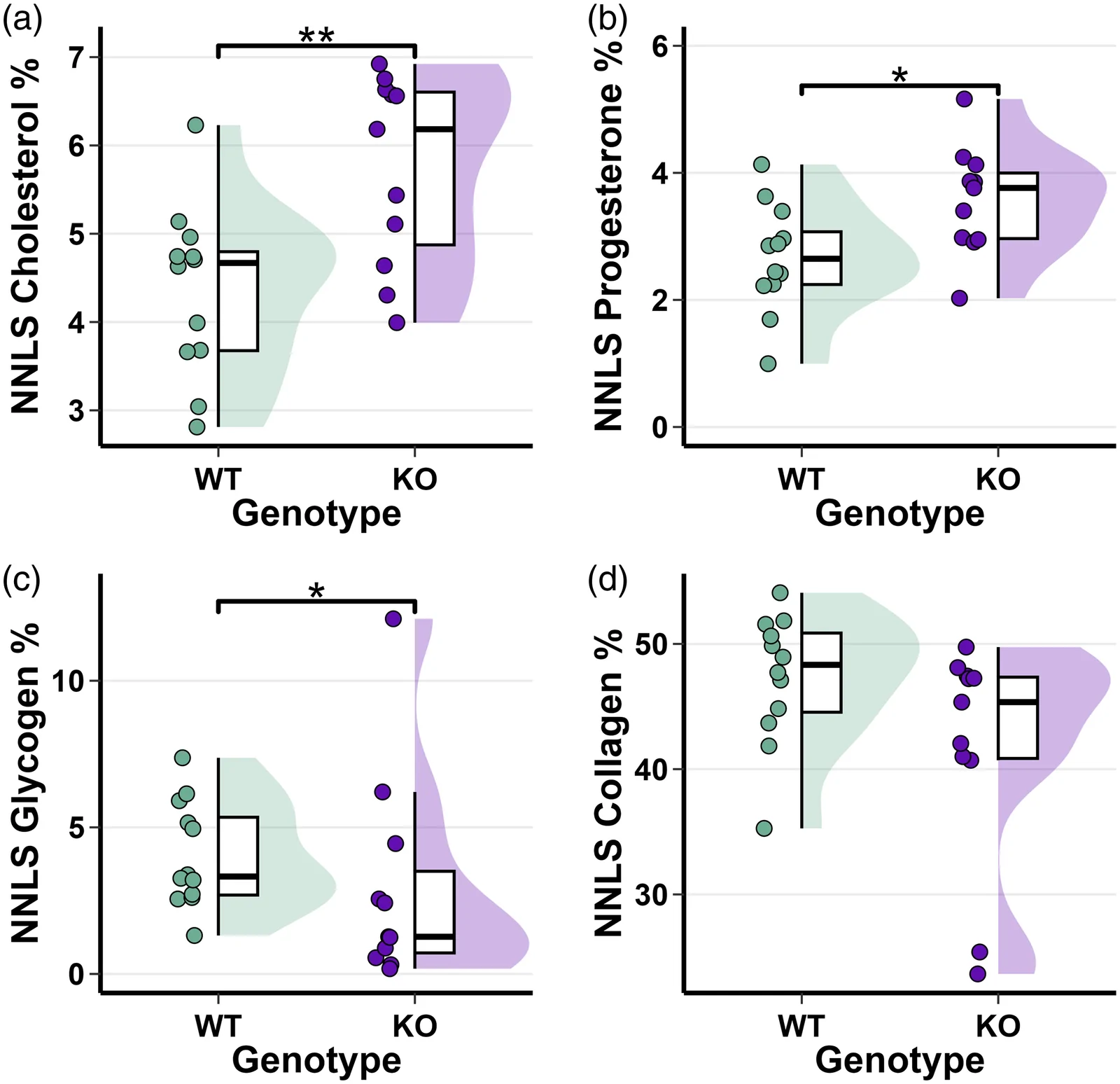
NNLS analysis of Raman spectra indicated significant changes in the knockout vagina compared with controls. The cholesterol (a) and progesterone (b) NNLS scores increased, whereas the glycogen NNLS score (c) decreased in knockout (KO, MOPQ=2/3, purple, n=11) mice when compared with wildtype (WT, MOPQ=0, green, n=12) controls. Alternatively, the collagen NNLS score (d) did not change significantly between the groups. Each excised (5×5  mm) ventral vaginal wall was subjected to 16 RS measurements in grid format [Fig. 1(a.3)], and the results averaged for one data point per mouse. Boxplot data is represented as the mean +/− interquartile range. **p<0.01, *p<0.05 denotes statistical significance. One knockout specimen was speckle coated prior to RS measurements, thus omitted from this section of analysis.

A supervised NNLS algorithm was used to quantify the concentration of pure component inputs on the acquired experimental spectra as a compliment to peak deconvolution. The cholesterol (p=0.0086) [Fig. 3(a)] and progesterone (p=0.015) [Fig. 3(b)] NNLS scores significantly increased in the knockout vagina when compared with the wildtype tissue. Glycogen NNLS coefficient significantly decreased (p=0.022) in the knockout mice when compared with their wildtype littermates [Fig. 3(c)]. No significant differences were detected in the collagen NNLS coefficients between the two groups (p=0.069) [Fig. 3(d)]. In addition, no significant differences were identified in the NNLS coefficients for water, estradiol, or lipid content (Fig. S7 in Supplementary Material).

### 3.2 Biomechanics

**Fig. 4:**
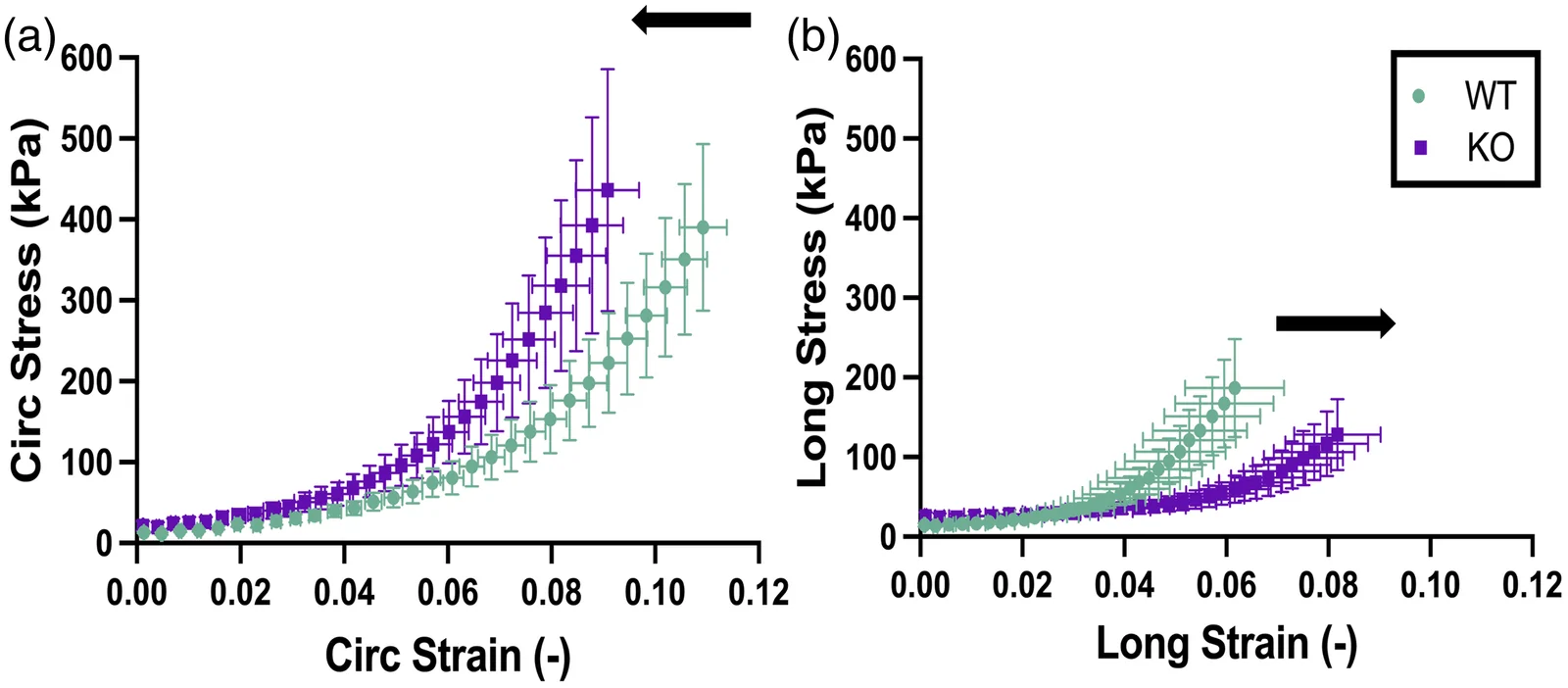
Circumferential (a) and longitudinal (b) stress-strain curves for the wildtype (WT, MOPQ=0, green circles) and knockout (KO, MOPQ=2/3, purple squares) murine vagina (n=12/group). Black arrows indicate the knockout tissue exhibited decreased circumferential extensibility and increased longitudinal extensibility compared with wildtype. Data represented as the mean +/− standard mean of error.

Compared with wildtype tissue, the knockout tissue exhibited direction-dependent alteration in mechanical response, specifically, knockout tissue showed decreased circumferential extensibility [Fig. 4(a)] and increased longitudinal extensibility compared with wildtype [Fig. 4(b)]. In wildtype tissue, the longitudinal direction was stiffer than the circumferential direction; however, this directional relationship was reversed in the knockout group (Fig. 4).

**Fig. 5:**
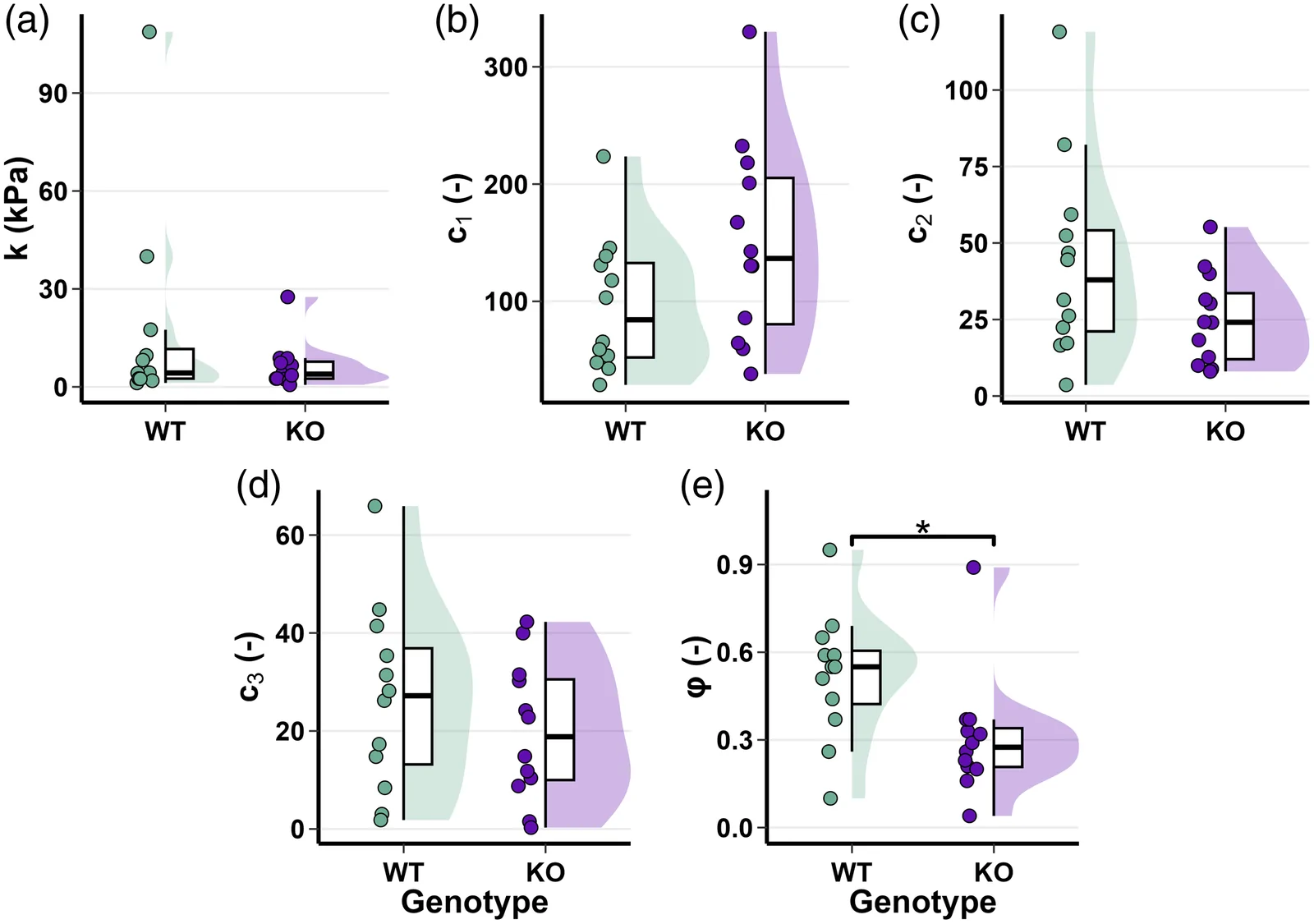
Mechanical parameters as calculated from the Fung constitutive model, in which no significant differences were identified for material parameters k (a), $c_{1}$ (b), $c_{2}$ (c), or $c_{3}$ (d); however, the anisotropy index (φ) significantly decreased in the knockout (KO, MOPQ=2/3, purple) mice when compared with their wildtype (WT, MOPQ=0, green) littermates (n=12/group) (e). Boxplot data are represented as the mean +/− interquartile range. *p<0.05 denotes statistical significance.

The vaginal tissues demonstrated a nonlinear mechanical behavior response under varying biaxial displacement ratios (Figs. S8-S9 in Supplementary Material). Further, the Fung constitutive model generated reasonable $R^{2}$ values, confirming acceptable model descriptions of the nonlinear biomechanical responses for both wildtype and knockout vagina (Figs. S8-S9 and Table S1 in Supplementary Material). Material parameter φ significantly decreased (p=0.011) in the knockout tissue, indicating increased anisotropy, or direction-dependence, when compared with their wildtype littermates [Fig. 5(e)] (Table 2). Other Fung parameters did not reach statistical significance; however, $c_{1}$, tentatively defined as the tissue’s resistance to changes in shape in the circumferential direction, visually increased (p=0.11) in the knockout group when compared with their wildtype littermates [Fig. 5(b)] (Table 2). Parameters $c_{2}$ (p=0.16) [Fig. 5(c)], or the tissue’s resistance to changes in shape in the longitudinal direction, $c_{3}$ (p=0.41) [Fig. 5(d)], or the mechanical interaction between the circumferential and longitudinal directions during loading, and k (p=0.63) [Fig. 5(a)], or the bulk resistance to changes in shape, were visually lower in the knockout vagina when compared with their wildtype littermates (Table 2).

**Table 2:** Mean and standard deviation for Fung constitutive model parameters and anisotropy index for murine vaginal tissues in wildtype and knockout vaginal samples.

Genotype		Model parameters					Anisotropy Index (φ)
		k (kPa)	$c_{1}$ (−)	$c_{2}$ (−)	$c_{3}$ (−)	$R^{2}$	
Wildtype	Mean	17.00	96.39	43.47	26.55	0.88	0.52
	SD	29.56	54.81	30.87	18.09	0.21	0.21
Knockout	Mean	6.40	150	25.43	19.88	0.98	0.31
	SD	6.90	81.48	14.37	13.54	0.01	0.20
*p* value		0.629	0.114	0.160	0.409		0.011*

* p<0.05 denotes statistical significance.

### 3.3 Histology

**Fig. 6:**
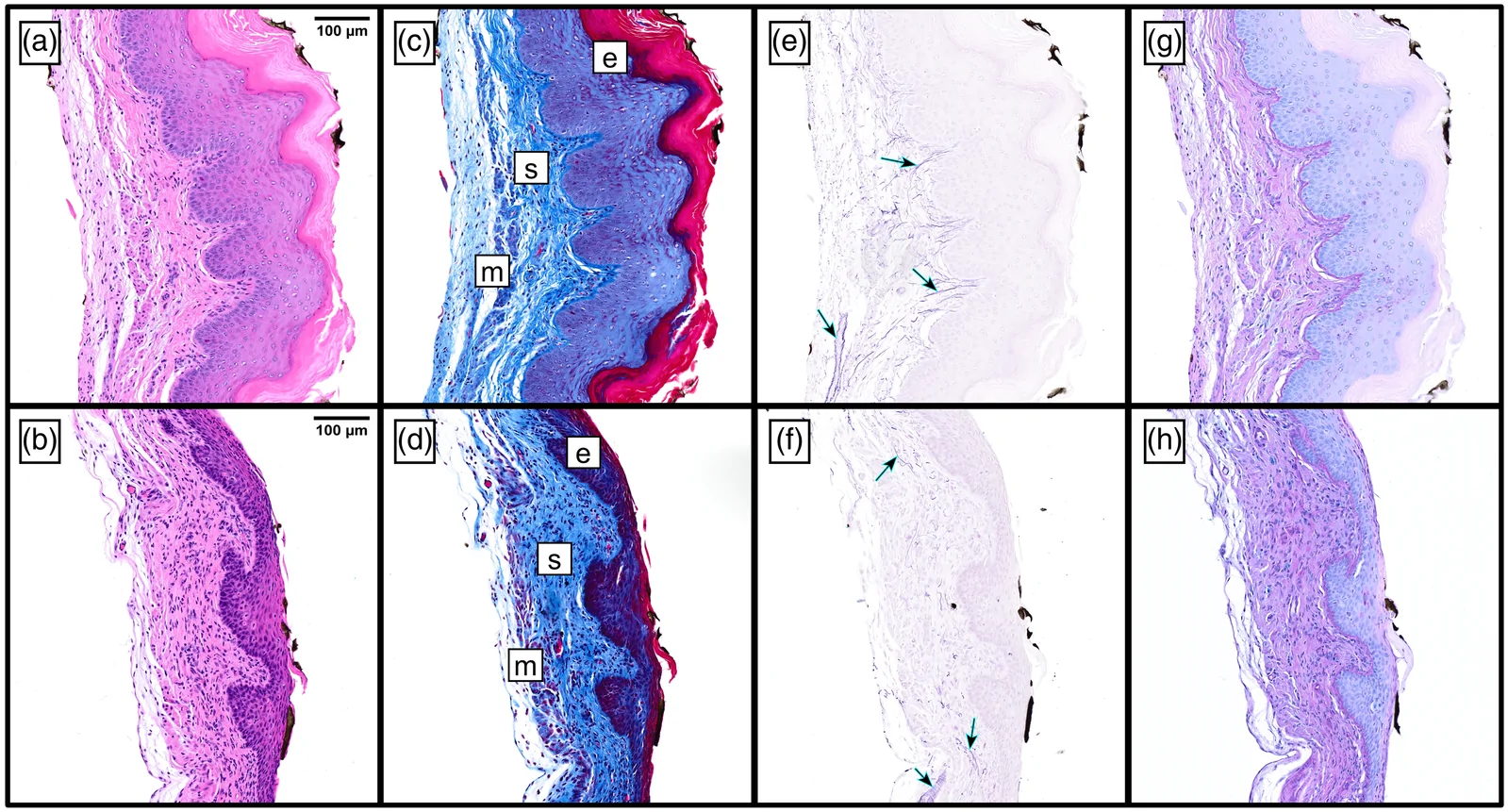
Representative histology of knockout and control vaginal tissues. Transverse histological regions of interest for wildtype (a, c, e, g) and knockout (b, d, f, h) vaginal samples. H&E (a, b) stained for nuclei (blue/purple) and extracellular matrix (pink/red). MTC (c, d) stained slides were used to differentiate tissue layers (m – muscularis, s – subepithelium, e – epithelium) and show nuclei (black), cytoplasm (red), and collagen (blue/green). For elastic fiber visualization, a modified Hart’s protocol (e, f) stained mature, functional elastic fibers (purple/black, indicated by arrows outlined in blue). Combined AB-PAS (g, h) stained for neutral carbohydrates (pink/red) and acidic carbohydrates (blue). Scale bar of 100  μm.

**Fig. 7:**
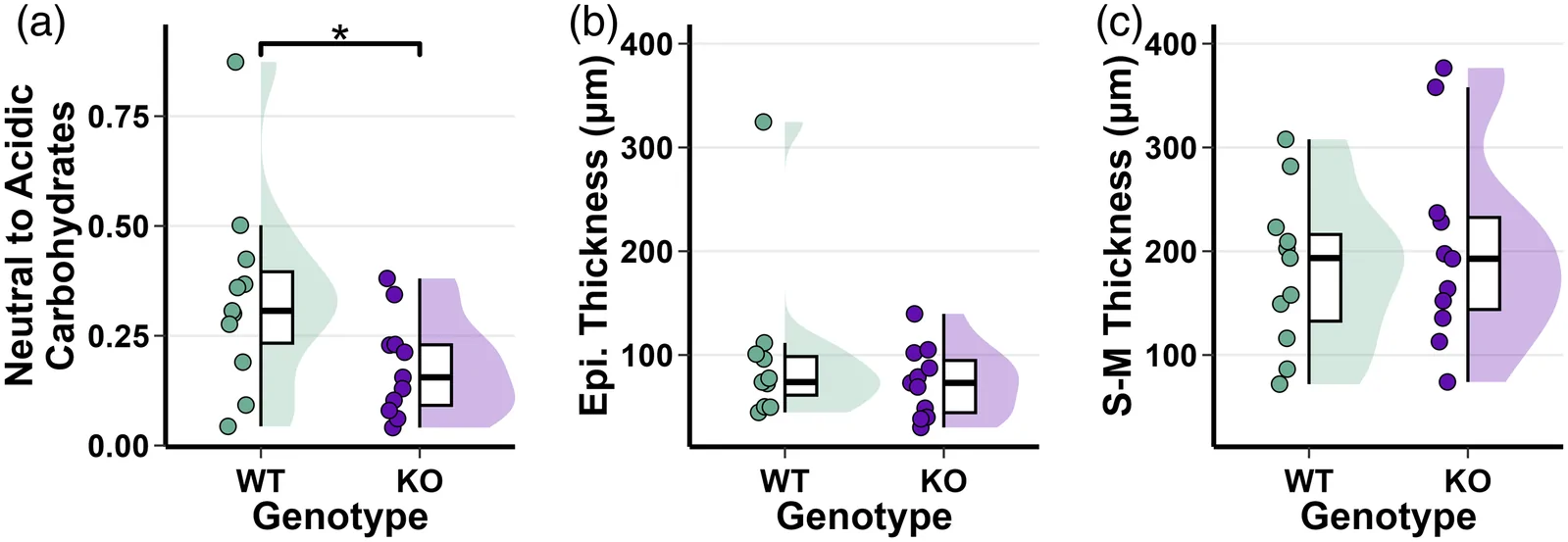
Histological analysis of AB-PAS-stained samples indicated variations with prolapse; however, histologically quantified thickness values did not differ. Neutral to acidic carbohydrate ratio (a) significantly decreased in the knockout (KO, MOPQ=2/3, purple) mice when compared with their wildtype (WT, MOPQ=0, green) littermates (n=11/group). Alternatively, epithelial thickness (b) and combined subepithelial-muscularis (S-M) thickness (c) in microns did not differ between the knockout (KO, MOPQ=2/3, purple) and wildtype (WT, MOPQ=0, green) vaginal samples (n=11/group). Boxplot data are represented as the mean +/− interquartile range. *p<0.05 denotes statistical significance. Two samples (n=1/genotype) were rendered incompatible with histological analysis and thus omitted.

**Fig. 8:**
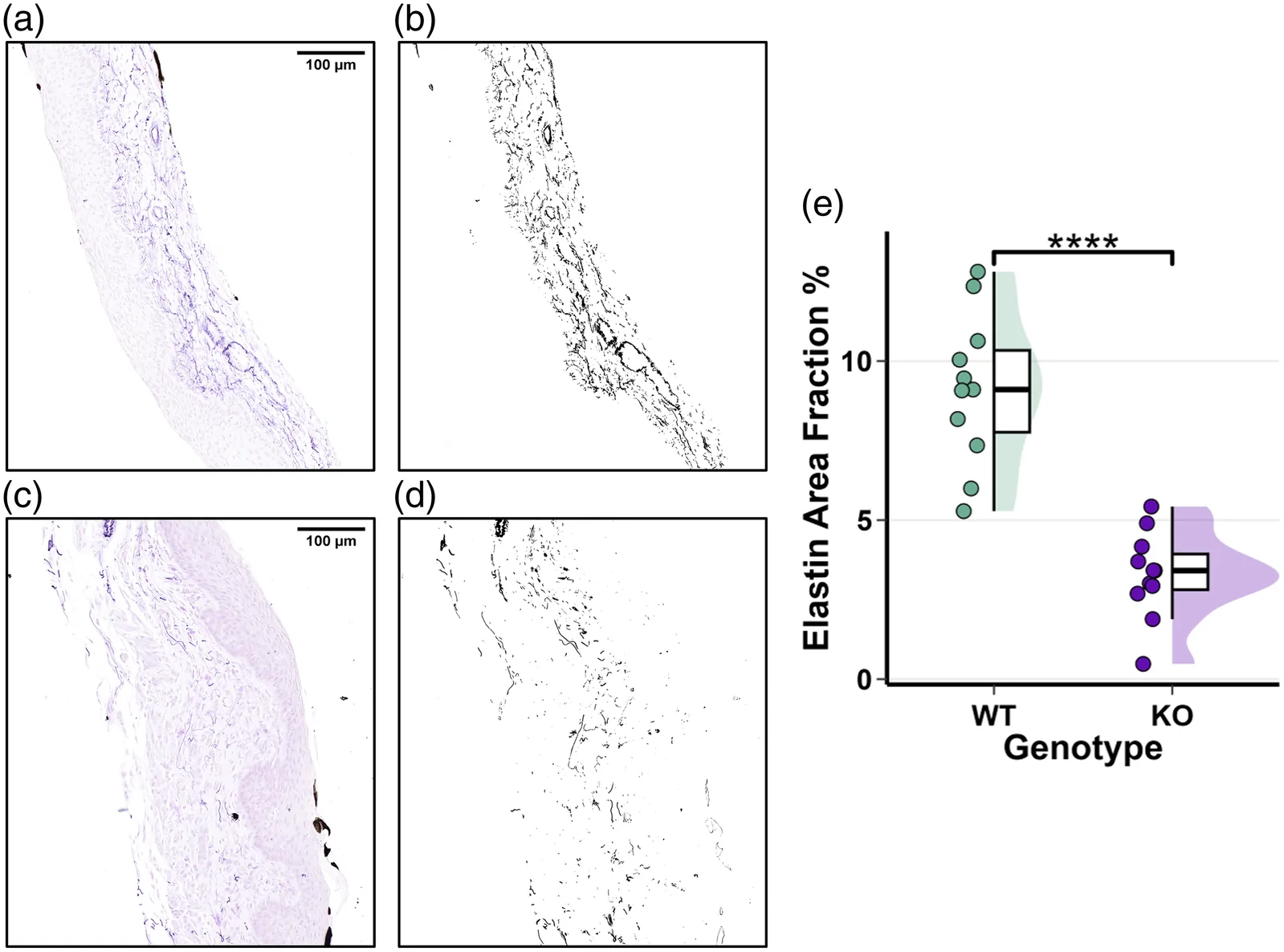
Representative vaginal sections for both wildtype (a, b) and knockout (c, d) vaginal samples. Slides were stained with Hart’s elastin (a, c) then imaged and processed to isolate the mature, functional elastic fibers (b, d) and calculate elastic fiber area fraction as a percentage of the subepithelium and muscularis. Elastic fiber area fraction significantly decreased in the knockout (KO, MOPQ=2/3, purple) mice when compared with their wildtype (WT, MOPQ=0, green) counterparts (n=11/group) (e). Boxplot data are represented as the mean +/− interquartile range. ****p<0.0001 denotes statistical significance. Scale bar of 100  μm. Two samples (n=1/genotype) were rendered incompatible with histological analysis and thus omitted.

Histological staining revealed distinct differences between the knockout and wildtype ventral vagina (Fig. 6). The ratio of neutral to acidic carbohydrates (calculated as the intensity ratio of red to blue channels from the AB-PAS stain, p=0.028) [Fig. 7(a)] as well as the area fraction of mature, functional elastic fibers (p<0.0001) (Fig. 8) significantly decreased in the knockout group when compared with their wildtype littermates. No other histology [Figs. 7(b)–7(c)] or laser micrometer (Fig. S10 in Supplementary Material) comparisons identified significant differences in tissue thickness.

### 3.4 Multimodal Correlation Analyses

**Fig. 9:**
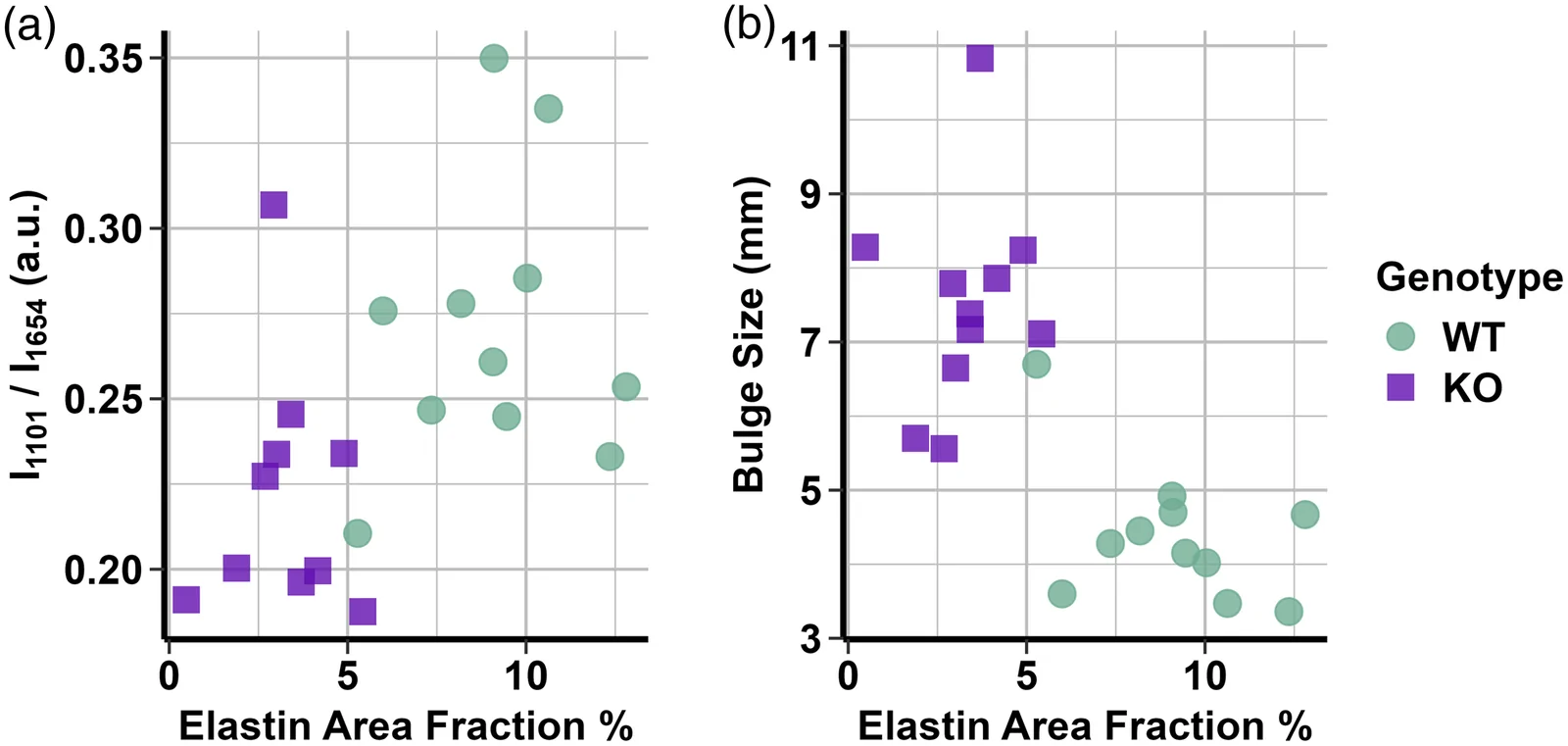
Scatter plots of variable pairings for Spearman correlation analyses. The RS-calculated crosslinked elastic fiber to ECM protein peak ratio ($I_{1101}/I_{1654}$) was positively correlated with the mature, functional elastic fiber area fraction from modified Hart’s elastin staining (a); however, there was a significant negative relationship between the bulge size and mature, functional elastic fiber area fraction (b) for both wildtype (WT, MOPQ=0, green circles, n=11) and knockout (KO, MOPQ=2/3, purple squares, $n=10^{†}\text{–}11^{‡}$) mice. Each excised (5×5  mm) ventral vaginal wall was subjected to 16 RS measurements in grid format [Fig. 1(a.3)], and the results averaged for one data point per mouse. One knockout specimen was speckle coated prior to RS measurements, thus not included in panel (a). Two samples (n=1/genotype) were rendered incompatible with histological analysis and omitted. ^†^Denotes panel (a) sample size, ^‡^denotes panel (b) sample size.

A Spearman correlation analysis demonstrated a statistically significant positive relationship between the $I_{1101}/I_{1654}$ crosslinked elastic fiber to ECM protein peak ratio and the mature, functional elastic fiber area fraction as identified via histology (p=0.0159, ρ=0.53) [Fig. 9(a)]. A significant negative association also existed between the bulge size in millimeters (increased bulge indicates more severe POP presentation) and the histologically quantified area fraction of mature, functional elastic fibers (p=0.0001, ρ=-0.74) [Fig. 9(b)].

## 4 Discussion and Conclusion

POP etiology remains widely unknown and understudied; however, we hypothesized ECM dysregulation may contribute to impaired tissue function. Currently, there are limited tools for tissue assessment without invasive and destructive approaches; therefore, our study assessed biochemical and biomechanical properties in vagina for knockout and wildtype age-matched mice using RS, planar biaxial, and histology methods. Further, correlative analyses across multiple complementary approaches strengthened the findings from RS compositional measurements as a potential surrogate or predictor for biomechanical changes with altered tissue compliance.

### 4.1 Optical Imaging Identified Variations in Murine Vaginal ECM Content With Prolapse

It is well established that elastic fibers in the $Fbln5^{-/-}$ mouse model are not assembled correctly; therefore, we hypothesized that the relative amount of functional elastic fibers would decrease in the knockout mice when compared with their wildtype littermates.^9^^,^^18^^,^^26^^–^^28^ In support of our hypothesis, nondestructive RS methods demonstrated a significantly decreased $I_{1101}/I_{1654}$ peak intensity ratio, representing the optical concentration of crosslinked elastic fibers to relative amounts of ECM proteins, in knockout mice when compared with their wildtype littermates [Fig. 2(e)]. In human skin, RS links increasing mechanical deformation to a decreasing $I_{1101}$, highlighting potential RS sensitivity to changes in elastic fiber loading in addition to formation of mature, functional elastic fibers.^100^ Specific analysis of the $I_{1101}/I_{1654}$ ratio revealed the RS peak $I_{1101}$ significantly decreased in the knockout vagina when compared with wildtype controls (Fig. S11B in Supplementary Material); however, the $I_{1654}$, indicative of protein secondary structure and abundance, did not change significantly (Fig. S11C in Supplementary Material). Validating our optical findings, Hart’s elastin area fraction significantly decreased in the knockout mice when compared with their wildtype littermates (Fig. 8) and positively correlated to both the nondestructive $I_{1101}/I_{1654}$ RS feature [Fig. 9(a)] and MOPQ measurement perineal bulge height [Fig. 9(b)]. Prior studies in the murine vagina investigating the impact of elastase on Hart’s elastin staining found a decreasing area fraction with elastic fiber degradation, indicating histological sensitivity to functional elastic fibers.^11^ Further, the decreased functional elastic fiber area fraction in the knockout mice reported herein is consistent with previously published literature in the $Fbln5^{-/-}$ vagina^18^ and aorta.^34^ Correlating histological elastic fiber area fraction to the $I_{1101}/I_{1654}$ peak ratio may allow for noninvasive monitoring of elastic fiber integrity with prolapse progression.

To test our hypothesis that collagen may compensate for decreased functional elastic fibers in the vagina, we investigated the $1003\;{\mathrm{cm}}^{-1}$ phenylalanine Raman peak. This mode persists in many ECM proteins; however, prior literature primarily implicates the $1003\;{\mathrm{cm}}^{-1}$ peak to collagens as phenylalanine is a precursory amino acid in collagen fiber synthesis.^83^^,^^101^^,^^102^ Further, the vaginal ECM is predominantly collagenous with a modest volume of elastic fibers and smooth muscle, suggesting this phenylalanine mode is likely representative of optical collagen content.^103^ The $1003\;{\mathrm{cm}}^{-1}$ RS peak is hypothesized to remain relatively unchanged with alterations in temperature and protein conformation and is commonly used as an internal standard for spectral normalization;^104^^,^^105^ however, the $1003\;{\mathrm{cm}}^{-1}$ AUC reported herein significantly increased in the knockout mice when compared with their wildtype littermates [Fig. 2(c)]. This may suggest that collagen is increasing in quantity to overcome the absence of functional elastic fibers with fibulin-5 deficiency; however, the spectral unmixing model did not confirm this as the NNLS collagen score trended down in the knockout mice when compared with their wildtype littermates [Fig. 3(d)]. This result was surprising; however, it may relate to the use of collagen I as a representative molecule for all ECM constituents (including elastin, actin, and collagens I and III) due to NNLS algorithm’s limitations as related to input variable multicollinearity (Figs. S2 and S3 in Supplementary Material). Alterations in specific collagen types implicated in tissue remodeling may further contribute to the nonsignificant NNLS collagen result.^18^ Further, the knockout vaginal tissue had decreased circumferential [Fig. 4(a)] but increased longitudinal [Fig. 4(b)] extensibility when compared with their wildtype littermates. These visual alterations in resistance to change in shape may relate to increased collagen crosslinking, orientation, and/or type; however, more research is needed to elucidate the specific microstructural relationship between collagen and POP.^41^^,^^106^

### 4.2 Knockout Vagina Exhibited Signs of Abnormal ECM Protein Organization

The murine vagina exhibited a nonlinear and direction-dependent stress-stretch response consistent with other soft biological tissues; however, the knockout vagina exhibited reversed directional extensibility when compared to their wildtype littermates (Fig. 4).^40^^,^^42^^,^^43^^,^^107^ It is well documented that tissue composition directly impacts its biomechanical function, wherein elastic fibers permit recoil and recovery while collagen provides tensile strength in the vagina.^8^^–^^10^ The implemented Fung model allows for robust comparisons of the calculated material parameters between the knockout and wildtype mice (Figs. S8-S9 in Supplementary Material). Herein, we have interpreted the material parameters as follows: $c_{1}$ and $c_{2}$ may represent the tissue’s resistance to circumferential and longitudinal deformations respectively, whereas $c_{3}$ may characterize the mechanical interaction of these directions.^45^^,^^88^ The parameter k reflects the tissue’s bulk resistance to change in shape.^45^^,^^88^ These model parameters were not significantly different across groups [Figs. 5(a)–5(d)]; however, the calculated anisotropy index φ, or the contribution of both circumferential and longitudinal directions to the tissue’s resistance to change in shape, significantly decreased in the knockout group when compared to their wildtype littermates [Fig. 5(e)]. Our compositional and histological analyses did not provide any direct correlations to explain this behavior, suggesting that additional tools to assess ECM structure and organization may provide insights into direction-dependent properties. It is important to note that the resting vaginal conformation resembles a cylinder folded in on itself.^108^ In $Fbln5^{-/-}$ mice, POP manifests as uterocervical and vaginal descent, causing the expansion of a conventionally collapsed cavity to accommodate the displaced organs and fundamentally altering vaginal loading conditions.^19^ In addition, the $Fbln5^{-/-}$ vagina at estimated physiological pressure operates at a higher state of biomechanical stress.^18^^,^^22^ Therefore, our results may indicate an increased directional preference and potential compensatory mechanism in the knockout vagina wherein the ECM undergoes remodeling and realignment as a protective measure against increased deformation.

### 4.3 Tissue Remodeling was Impaired in the Knockout Murine Vagina

In addition to changes in the load-bearing components, we hypothesized inflammatory ECM alterations due to the role of fibulin-5 in MMP regulation.^15^^,^^16^^,^^20^^,^^63^^–^^65^ Specifically, MMP-9 is a known component involved in ECM-degradation associated with chronic inflammation that increases in the murine vagina with POP.^15^^,^^66^^–^^68^ The body’s immune response is complex and recruits multiple biological components that may result in dysregulated lipid metabolism.^109^ To this end, the $I_{1300}/I_{1246}$ peak ratio, tentatively identified as the lipid to protein ratio, increased in the knockout murine vagina when compared with their wildtype littermates [Fig. 2(b)]. Lipids are critical components in the vagina that contribute to ECM homeostasis wherein pathophysiological processes, such as inflammation, alter overall lipid content.^110^^,^^111^ In patients with vulvovaginal candidiasis and cytolytic vaginosis there is an increase in concentration of lipids related to apoptosis and chronic inflammation such as phospholipids and palmitic acid, indicating that a relationship may exist between lipid profile and pathological vaginal microenvironment disruption.^111^ Deeper analysis into the $I_{1300}/I_{1246}$ peak ratio highlighted a significant increase in $I_{1300}$ lipid peak (Fig. S11C in Supplementary Material); however, no changes were observed in the $I_{1246}$ protein peak (Fig. S11B in Supplementary Material) in the knockout mice when compared to their wildtype littermates. In support of this finding, the cholesterol NNLS score increased significantly in the knockout mice with respect to their wildtype littermates [Fig. 3(a)]. Dysregulated cellular cholesterol metabolism is linked to pathologies in various organ systems, such as Parkinson’s,^112^^,^^113^ systemic lupus erythematosus,^114^ and cancer^115^ through increases in MMPs indicative of inflammation.^116^ Cholesterol content in vaginal fluid is known to fluctuate as a function of menstruation; however, all mice used in this study were sacrificed at estrus.^117^ Therefore, the increased cholesterol NNLS score in knockout mice may indicate dysregulated cholesterol metabolism and increased inflammation with POP, potentially relating to impaired ECM remodeling.

Glycogen is a polysaccharide used for glucose storage and tissue repair found in high quantities in the liver and skeletal muscle; however, it is also present in other tissues.^118^ Hormones are thought to influence luminal glycogen deposits wherein glycogen content decreases significantly in the menopausal ewe^99^ and human^119^ vagina and murine uterus with reproductive aging.^62^ To this end, the glycogen NNLS score significantly decreased in the knockout mice when compared to their wildtype littermates [Fig. 3(b)]. Supporting the label-free RS results, the ratio of neutral (pink/red, glycogen) to acidic (blue) carbohydrates (extracted from histological analysis of AB-PAS-stained murine vaginal tissue) decreased in the knockout mice when compared with the wildtype littermates [Fig. 7(a)]. The vagina produces and stores glycogen to strengthen epithelial barrier defense as well as provide cellular nutrients for the subepithelial and muscularis layers.^120^^–^^122^ Prior literature in pulmonary fibrosis reports increases in glycogen synthesis and degradation in diseased fibroblasts which may indicate a correlation between ECM fiber deposition and glycogen depletion.^123^ Herein, we hypothesize the decreased glycogen content observed in the knockout murine vagina when compared with their wildtype littermates may relate to dysregulated ECM remodeling; however, further research into glycogen’s impact on the vagina with POP is needed.

Progesterone is a sex hormone produced in the ovary that is critical to vaginal tissue homeostasis.^124^^,^^125^ Progesterone levels are known to relate to changes in vaginal epithelial cell phenotype in ovariectomized rats with implications in cervical tissue stiffening and ECM remodeling in mice.^124^^,^^125^ Herein, the progesterone NNLS score significantly increased in the knockout murine vagina when compared with their wildtype littermates [Fig. 3(b)]. Prior research in the murine cervix relates progesterone treatment to *Col1A1* and *Eln* expression as well as ECM microstructure.^125^ Herein, the increased progesterone NNLS score may relate to the decreased anisotropy index (φ), where the elevated progesterone content may alter collagen fiber alignment as previously shown in the murine cervix.^125^ It is well-established that systemic steroid hormones fluctuate between and within murine estrous cycle stages.^126^ Specifically, progesterone and estrogen trend downward from early to late estrus which may contribute to the observed differences; however, variations in systemic and tissue level sex hormone content in the $Fbln5^{-/-}$ mouse remain unknown. All animals used in this study were age-matched and sacrificed at estrus to control systemic hormone profiles; however, genotype specific differences in steroid hormone levels remain undefined in this model. Future research should implement additional methods such as steroid immunoassays or liquid chromatography–tandem mass spectrometry to elucidate the impact of systemic and local progesterone and estrogen on vaginal ECM organization, composition, and function in the $Fbln5^{-/-}$ mouse to discern hormonal influences on POP presentation.^127^

### 4.4 Prolapse Did Not Alter Tissue Thickness

One of the major clinical risk factors of POP is chronological age which is confounded by the onset of menopause, or the cessation of ovarian function that leads to systemic estrogen decline.^128^^,^^129^ However, studies relating menopause and POP are conflicting, with some reporting a direct association between menopause and POP severity,^40^^,^^130^^,^^131^ whereas others claim no significant correlation.^132^ Upwards of 50% of postmenopausal women experience genitourinary syndrome of menopause, including vaginal wall atrophy and inflammation as a result of decreased estrogen.^129^ The multicollinearity between chronological age and hormonal status may contribute to these conflicting reports as atrophy is a symptom of menopause which occurs with aging,^132^^–^^134^ whereas a major POP risk factor is age itself.^128^^,^^135^ Further, vaginal wall thinning in an ewe model of menopause directly correlates to decreasing glycogen content; however, literature in the $Fbln5^{-/-}$ mouse report no significant differences in the knockout vaginal wall thickness when compared with the wildtype controls.^18^^,^^99^ Mice are a well-established and robust option for age-related studies due to their comparatively short lifespan; however, they do not naturally experience a hormonal equivalent of human menopause with age.^136^^,^^137^ To this end, no significant differences were detected in the knockout vaginal wall thickness when compared with their wildtype littermates [Figs. 7(b)–7(c) and Fig. S10 in Supplementary Material]. These results may indicate that vaginal atrophy and POP are separate events that each co-occur with increased chronological age in the $Fbln5^{-/-}$ murine POP model. Future research in the $Fbln5^{-/-}$ mouse wherein menopause is induced via ovariectomy is needed to enable the decoupling of hormone reduction and age.

### 4.5 Experimental Limitations and Future Directions

Despite efforts to control relevant variables, this study was not without limitations. Although the mouse is an effective laboratory model, the loading conditions of the reproductive organs are different than in the bipedal human. In addition, the $Fbln5^{-/-}$ mouse spontaneously develops prolapse as a function of chronological age and regardless of parity; however, in women the risk factors are multifaceted and much more complex.^19^^,^^128^ This study used a previously reported C57BL/6J mouse lineage with expected $Fbln5^{-/-}$ genotype and analogous phenotype.^138^ Additional studies were based on the C57BL/6 x 129SvEv background,^15^^,^^16^^,^^18^^,^^19^^,^^22^^,^^23^^,^^26^^,^^139^ which may contribute to observed differences. Nevertheless, this murine model allows for the evaluation of biochemical and biomechanical aspects of POP pathophysiology in months compared with years in humans while providing access to tissue samples and permitting isolation of one confounding factor at a time.

Another limitation of this study was the NNLS algorithm for Raman spectral unmixing as the assumption of input variable independence eliminated pure components of a similar biochemical makeup from model consideration. Further, the input variables constrain the model to a specific shape for each pure component, preventing deeper analysis into minute spectral changes in line shape or other phenomena that accompany protein denaturation and microstructural changes.^140^ Although not an exact depiction of ECM changes in POP, the NNLS model represents a complementary approach to peak deconvolution and a critical first step to understanding the biochemical changes in the vagina related to prolapse. Further, the histology presented herein has provided orthogonal support of some NNLS modeling results; however, future work validating additional model components such as lipid content will require cryopreservation protocols. Additional research investigating alternate spectral modeling methods that can include and differentiate relevant ECM constituents is necessary to improve interpretability and assist in treatment research.

The presented study represents a critical first step toward noninvasive optical characterization of POP in the $Fbln5^{-/-}$ mouse model. Further, this work exhibits significant translational potential for longitudinal monitoring in animal models of POP; however, more research is needed to determine measurement outcomes with respect to the intact murine anatomy and the current RS probe design. In addition, investigations into spatial variability in the murine vagina may provide critical insight into POP pathophysiology. The proposed technology could serve as a clinical early diagnosis tool, correlating optical biomarkers of composition to the disease’s composition-function relationship before symptom manifestation.

In summary, this study quantified biochemical, histological, and biomechanical changes in a knockout murine model of POP and compared them to aged-matched wildtype littermates. These nondestructive and label-free measurements of tissue ECM composition correlated with histological properties in a mouse model of POP. Further, we have presented an established and verified workflow for nondestructive compositional analysis with future implications in noninvasive clinical POP evaluation and treatment monitoring. A future direction will serve to extend this research to human subjects for technological validation to establish this approach as a viable clinical tool for patient tissue assessment.

## Supplementary Material

10.1117/1.BIOS.3.3.032106.s01

## Data Availability

The experimental data that support the findings of this publication are available in the Texas Data Repository (https://doi.org/10.18738/T8/TUW5NY).
