# Supplementary material for: Raman spectroscopy for nondestructive detection of vaginal tissue alterations in the fibulin-5 murine model of pelvic organ prolapse
Source: Biophotonics Discov. 2026 Aug 25;3(3):032106. doi: 10.1117/1.BIOS.3.3.032106 (PMC13557458; doi:10.1117/1.BIOS.3.3.032106)
Supplement: Supplementary file 1 [file BIOS_003_032106_SD001.pdf]

Raman spectroscopy for non-destructive detection of vaginal tissue alterations in the fibulin-5 murine model of pelvic organ prolapse

Triniti N. Vanoven,[a,b] Fatemeh Vahidi Zaman, MS,[a,b] Evelyn T. Pan, MD,[c] Jessica K. Baas, MS,[a] Maria E. Florian Rodriguez, MD,[a,b,c,d] Kristin S. Miller, PhD,[a,b,c,e] Isaac J. Pence, PhD,[a,b,f,g,*]

[a] University of Texas Southwestern Medical Center, Department of Biomedical Engineering, Dallas, United States of America
[b] University of Texas at Dallas, Department of Bioengineering, Richardson, United States of America
[c] University of Texas Southwestern Medical Center, Department of Obstetrics and Gynecology, Dallas, United States of America
[d] University of Texas Southwestern Medical Center, Cecil H. and Ida Green Center for Reproductive Biology Sciences, Dallas, United States of America
[e] University of Texas at Dallas, Department of Mechanical Engineering, Richardson, United States of America
[f] University of Texas Southwestern Medical Center, Charles and Jane Pak Center for Mineral Metabolism and Clinical Research, Dallas, United States of America
g University of Texas Southwestern Medical Center, Department of Internal Medicine, Dallas, United States of America

*Corresponding Author, E-mail: Isaac.Pence@UTSouthwestern.edu

A. Biomechanical Data Analysis

Sample deformation was quantified using a MATLAB-based digital image correlation framework as previously described [90]. Briefly, the deformation gradient tensor ($\mathbf{F}$), was computed from the displacements of reference points tracked near the center of each specimen, assuming homogeneous deformation where strain is considered uniform over a small, centralized region. The Green–Lagrange strain tensor ($\mathbf{E}$) was then obtained as [41, 45, 89]:

$$\mathbf{E} = \frac{1}{2}(\mathbf{F}^T \mathbf{F} - \mathbf{I}), \qquad (1)$$

where $\mathbf{F}^T$ is transpose deformation gradient tensor and $\mathbf{I}$ is identity tensor.

Under planar biaxial loading, the first Piola–Kirchhoff stresses ($P_{\theta\theta}$, P_{zz}) were computed from the measured loads [41, 45, 89], where cylindrical coordinates (θ, z) were used to align with the vaginal wall's native circumferential (θ) and longitudinal (z) axes:

$$P_{\theta\theta} = \frac{T_{\theta\theta}}{A_{\theta\theta}}, \qquad P_{zz} = \frac{T_{zz}}{A_{zz}}. \qquad (2)$$

Where $T_{\theta\theta}$ and T_{zz} are the current circumferential and longitudinal loads measured by the in-line load cells, and $A_{\theta\theta}$ and A_{zz} are the corresponding undeformed (original) cross-sectional areas [89]. The in-plane first Piola–Kirchhoff stress tensor ($\mathbf{P}$) was represented as [41]

$$\mathbf{P} = \begin{bmatrix} P_{\theta\theta} & 0 \\ 0 & P_{zz} \end{bmatrix}. \qquad (3)$$

To characterize nonlinear tissue behavior, the following Fung exponential strain-energy function ($W(\mathbf{E})$) was implemented [41, 141]

$$W(\mathbf{E}) = \frac{k}{2}(e^{Q(\mathbf{E})} - 1), \quad (\mathbf{4})$$

$$Q(\mathbf{E}) = c_1 E_{\theta\theta}^2 + c_2 E_{zz}^2 + 2c_3 E_{\theta\theta} E_{zz}. \quad (\mathbf{5})$$

Here parameter k represents a stress-like scaling factor controlling the bulk tissue resistance to changes in shape in kPa. $Q(\mathbf{E})$ characterizes the anisotropic contribution of the tissue arising from the underlying extracellular matrix composition and organization, such as collagen fiber architecture, while $\boldsymbol{E_{\theta\theta}}$ and $\boldsymbol{E_{zz}}$ correspond to the Green–Lagrange strains in the circumferential and longitudinal directions, respectively [88].

The dimensionless model parameters c_1 and c_2 are hypothesized to describe the tissue's resistance to changes in shape in the circumferential and longitudinal directions, respectively, for extracellular matrix components such as collagen fibers, whereas c_3 describes the coupling interaction between directions, which may reflect the influence of stretch in one direction on the mechanical response in the other or microstructural phenomena such as interactions between collagen fibers, cross-links, or other extracellular matrix component interactions [45, 88]. The theoretical first Piola–Kirchhoff stress ($\mathbf{P}^{th}$) was obtained by taking the derivative of the pseudostrain energy function with respect to the Green–Lagrange strain [45, 142]:

$$\mathbf{P}^{th} = \mathbf{F}\frac{\partial W(\mathbf{E})}{\partial \mathbf{E}} \quad (\mathbf{6})$$

Model parameters (k, c_1, c_2, c_3) were determined for each sample by using a nonlinear regression to calculate the best-fit values to minimize the least-squares difference between theoretical and experimental ($\mathbf{P}^{exp}$) stress values across the final loading cycle of all three protocols using the following objective function [45, 143].

$$\min_{\theta} \sum_{k} \left[\left(P_{\theta\theta,k}^{th} - P_{\theta\theta,k}^{exp}\right)^2 + \left(P_{zz,k}^{th} - P_{zz,k}^{exp}\right)^2 \right], \qquad \theta = (k, c_1, c_2, c_3) \quad (\mathbf{7})$$

The calculated R^2 evaluates the model's predictions with respect to the experimental stress data. R^2 values closer to one indicate a better fit, confirming the model's accuracy. Lastly, φ is an index of material anisotropy and quantifies the contribution of each direction to tissue resistance to changes in shape based on the identified model parameters: φ closer to one indicates highly isotropic materials and closer to zero demonstrates higher anisotropic (direction-dependent) materials [45, 88, 141, 144].

$$\varphi = \min\left(\frac{c_1 + c_3}{c_2 + c_3}, \frac{c_2 + c_3}{c_1 + c_3}\right) \tag{8}$$

B. Supplemental Figures

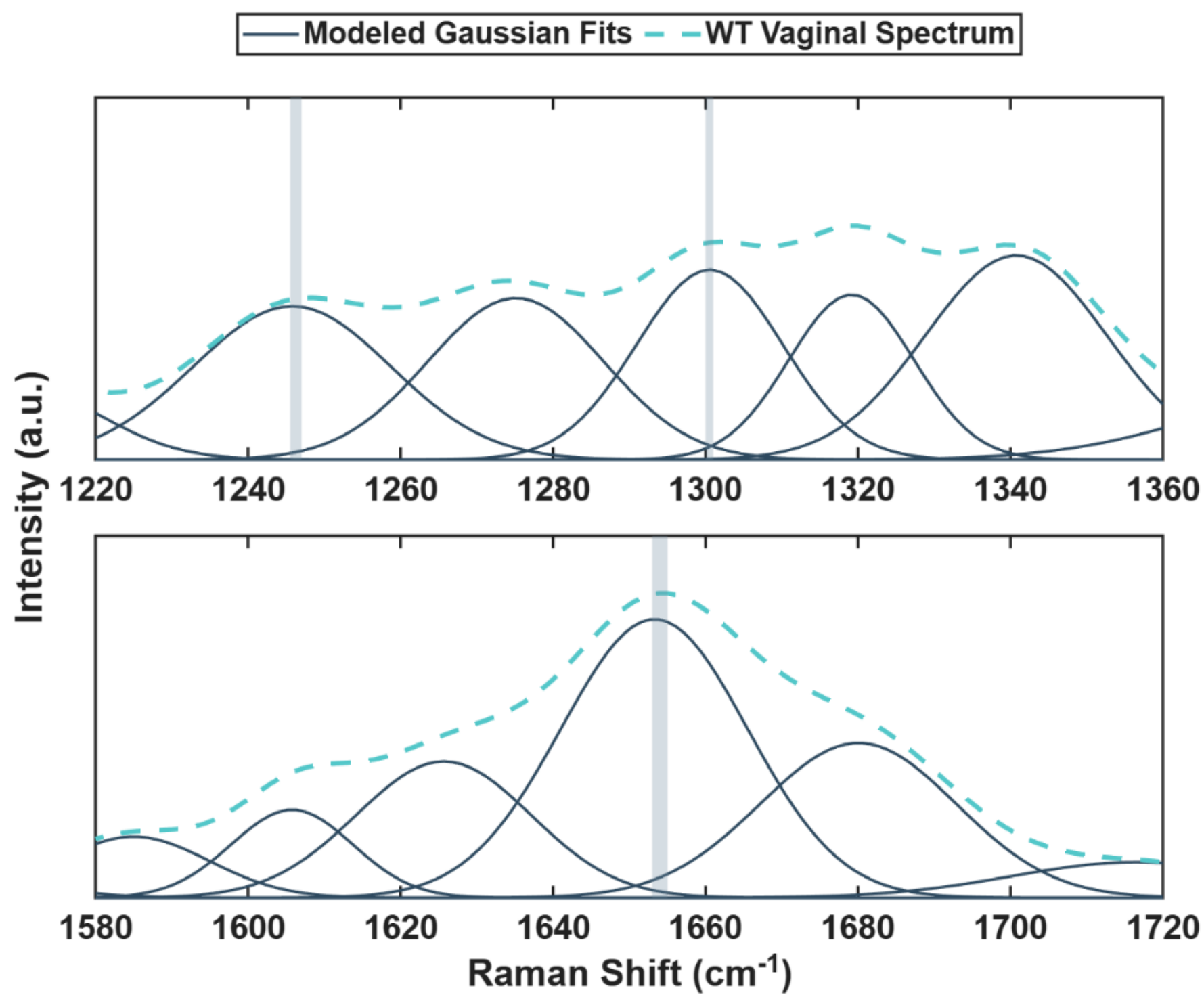

Figure S1. A representative vaginal spectrum (WT, MOPQ=0, light blue dashed line) was fit with five and six Gaussian curves (dark blue solid lines) in the amide III (**A**) and amide I (**B**) regions, respectively. Highlighted in grey are identified peaks of interest at 1246cm^{-1} (± 0.72cm^{-1}), 1300cm^{-1} (± 0.32cm^{-1}), and 1654cm^{-1} (± 0.40cm^{-1}) with tentative peak assignments in Table 1. Additional peaks were fit corresponding to established vibrational modes of ECM constituents such as 1276cm^{-1} (amide III), 1320cm^{-1} (CH_3/CH_2 twisting), 1340cm^{-1} (CH_3/CH_2 wagging), 1584cm^{-1} (C=C olefinic stretch), 1605cm^{-1} (C=C bending), 1625cm^{-1} (tryptophan), and 1680cm^{-1} (amide I β-sheet) [55-57, 59, 74, 145].

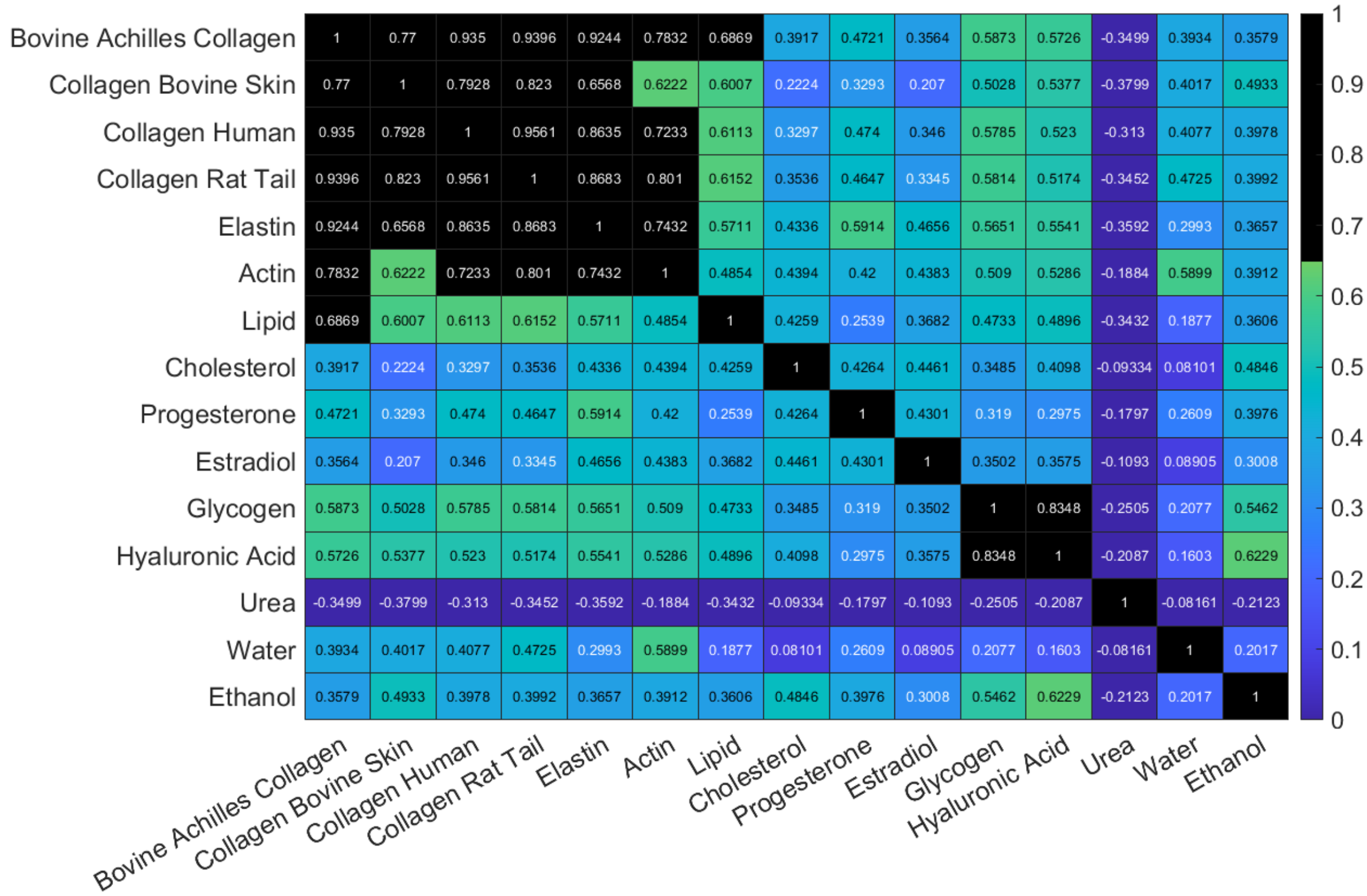

Figure S2. Spearman correlation analysis heatmap of potential pure component inputs for the NNLS model. Black boxes indicate a correlation coefficient greater than the defined threshold of ρ > 0.65.

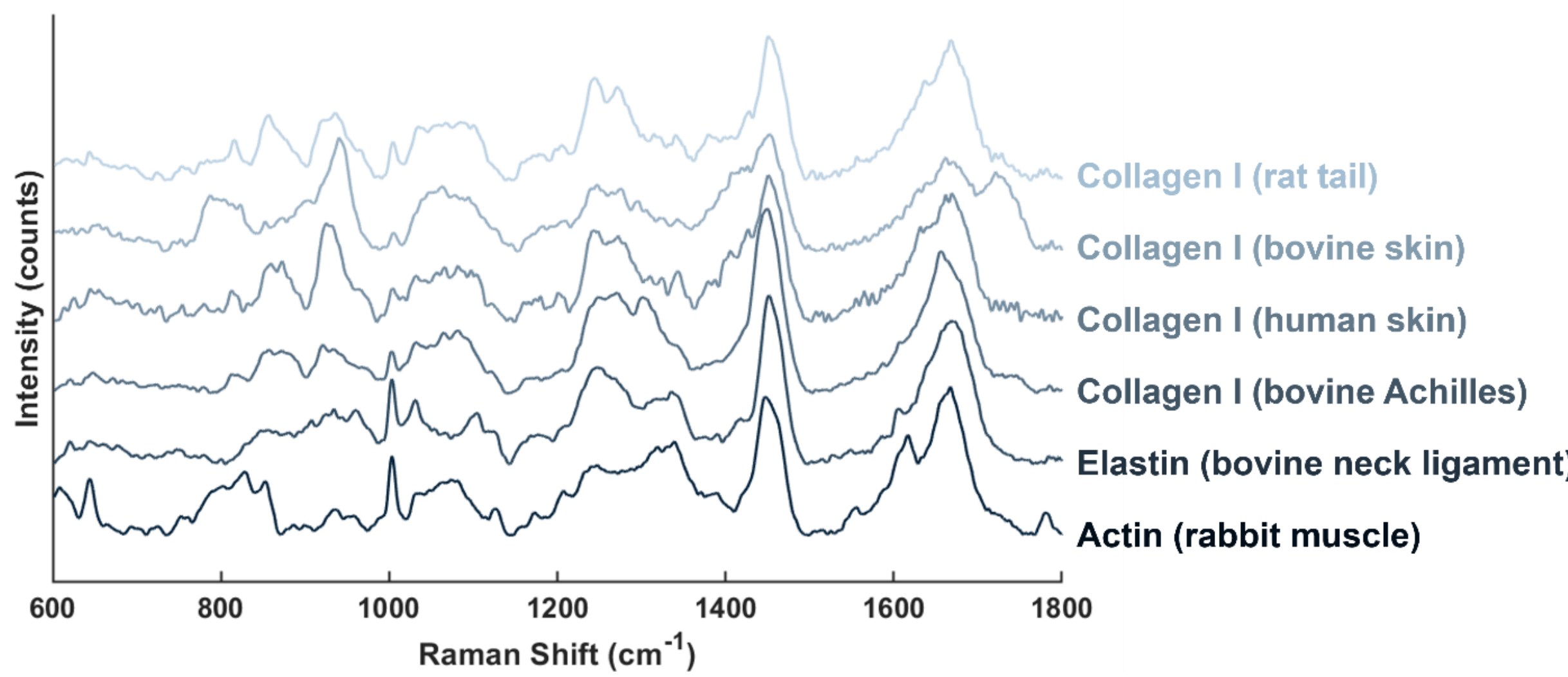

Figure S3. Raman spectra of collagen I from varying origins (rat tail, bovine skin, human skin, and bovine Achilles tendon) as well as elastin and actin. Previously, we published elastin as the representative ECM protein in the aging murine uterus; however, for the vagina, rat tail collagen I was more a more appropriate example [62].

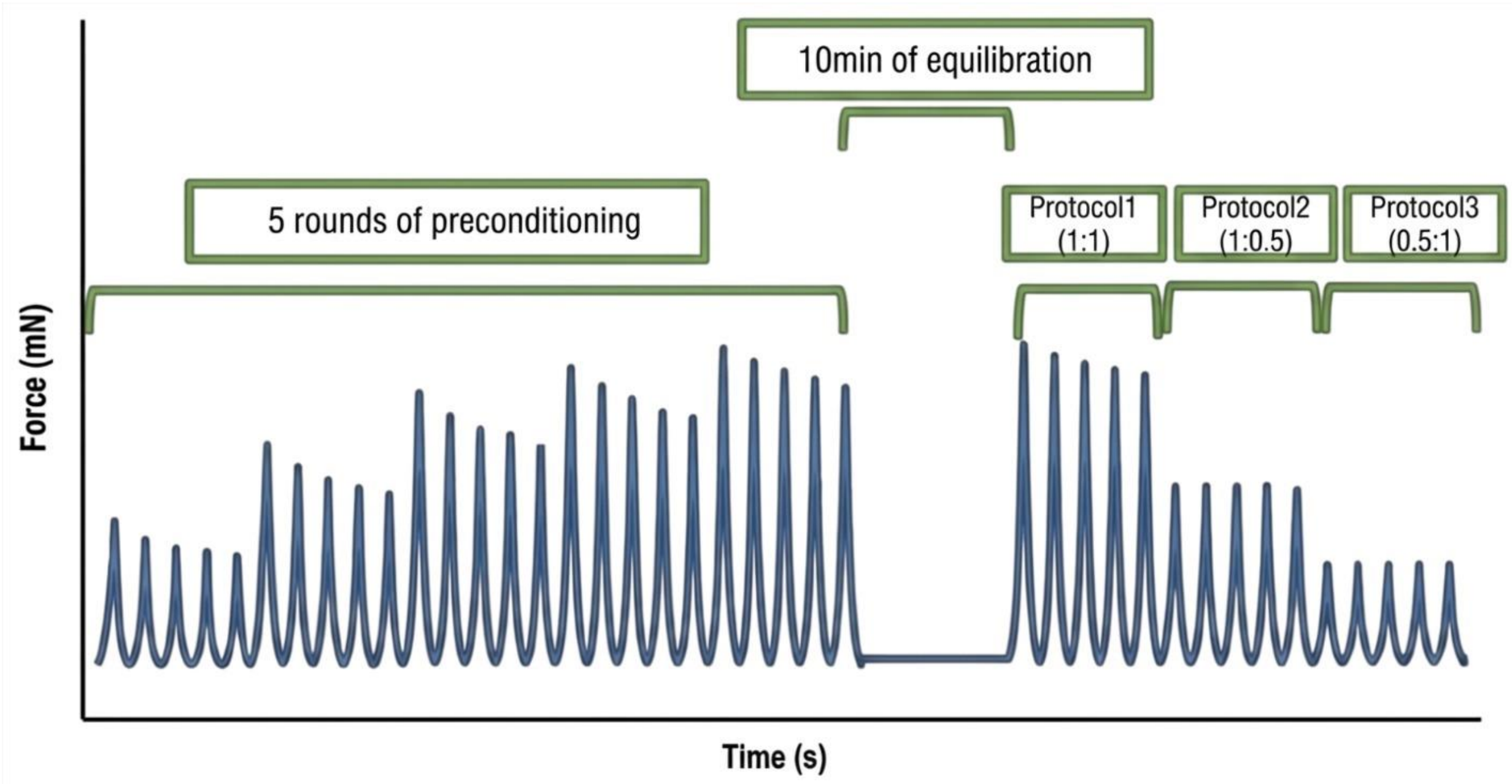

Figure S4. Representative force–time curves from the biaxial mechanical testing protocol. The tissue underwent minimum five rounds of preconditioning followed by a 10-minute equilibration period [45]. Then, three loading unloading protocols (displacement ratios of 1:1, 1:0.5, and 0.5:1) were applied sequentially. Some figure components were made in BioRender.

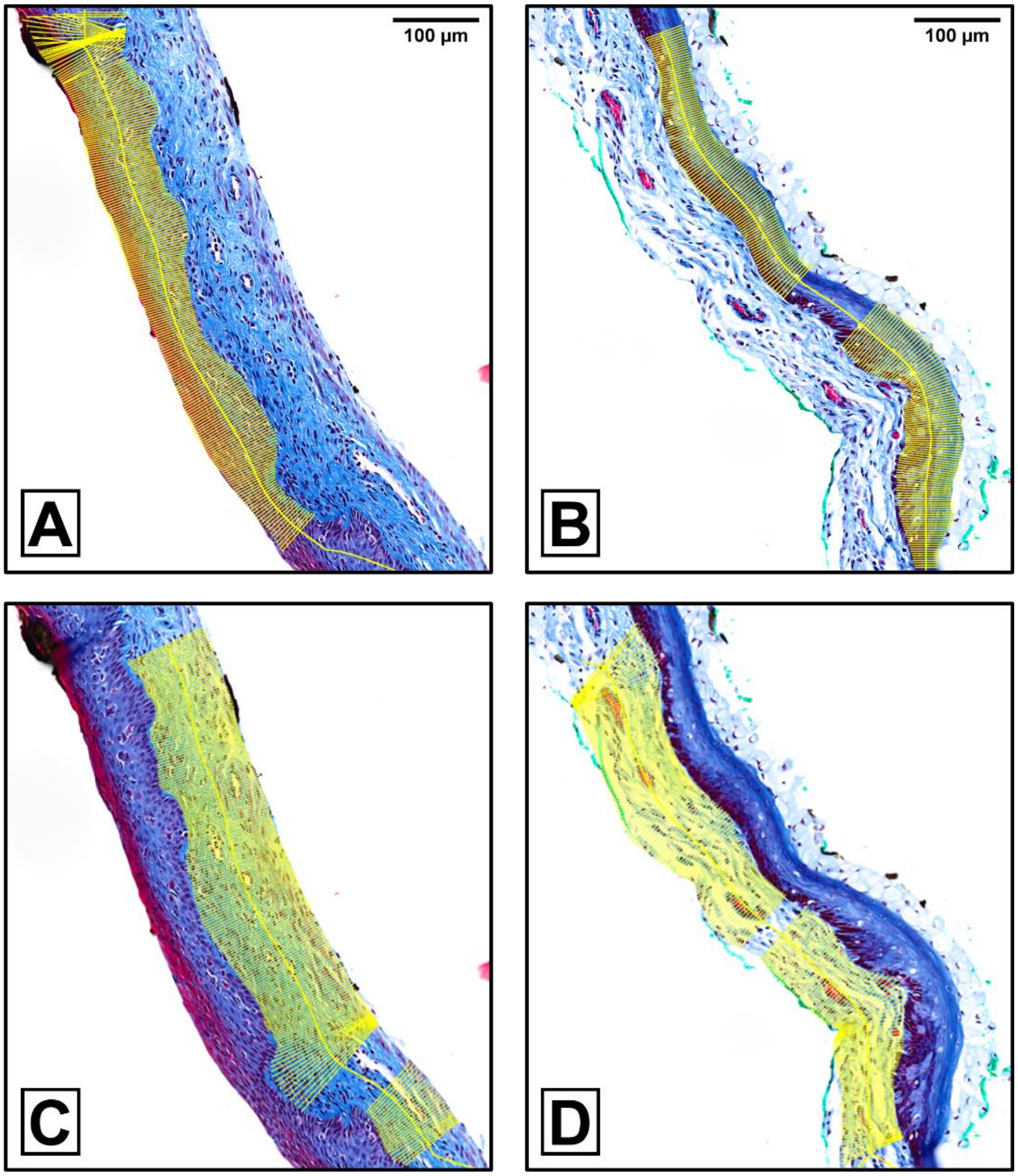

Figure S5. Representative histological regions of interest for wildtype (**A**, **C**) and knockout (**B**, **D**) vaginal samples. The modified ThicknessTool code draws multiple vectors in the defined tissue layer then outputs the average thickness across the region of interest [97]. Images depict both the epithelial (**A**, **B**) and combined subepithelial-muscularis (**C**, **D**) layer thicknesses. Scale bar of 100µm.

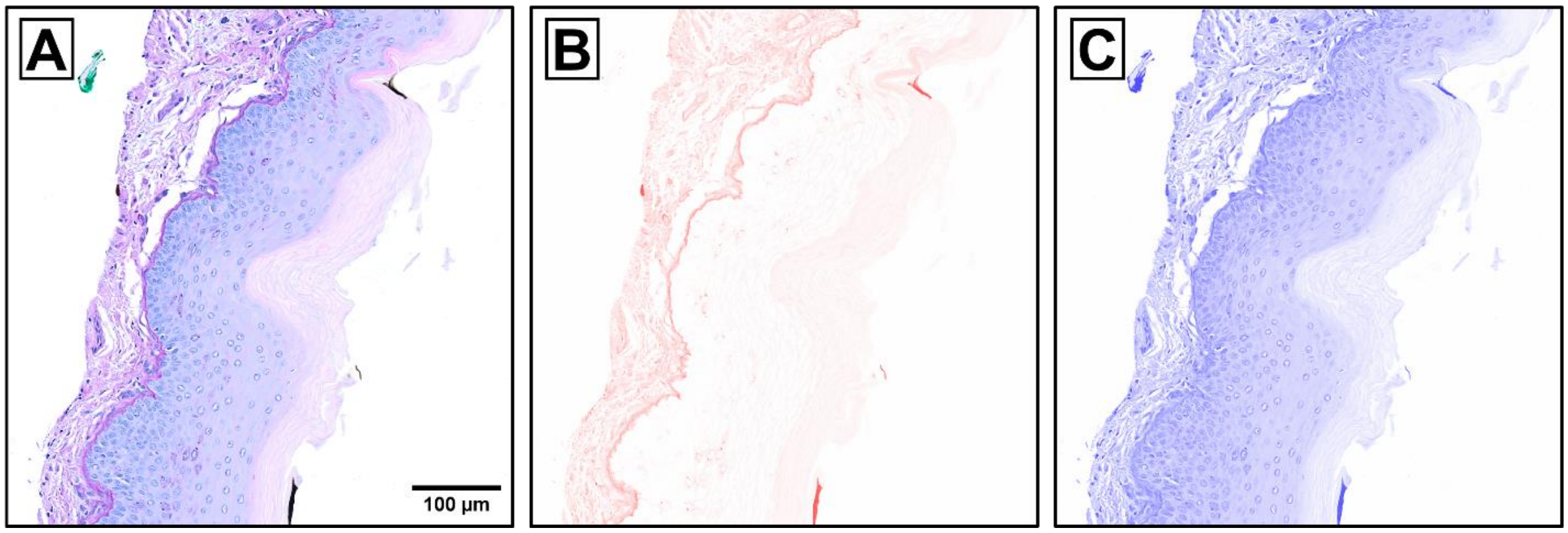

Figure S6. Combined AB-PAS (**A**) stained for neutral carbohydrates (pink/red) and acidic carbohydrates (blue). The image was unmixed and the red (**B**) and blue (**C**) channels used for analysis. Scale bar of 100µm.

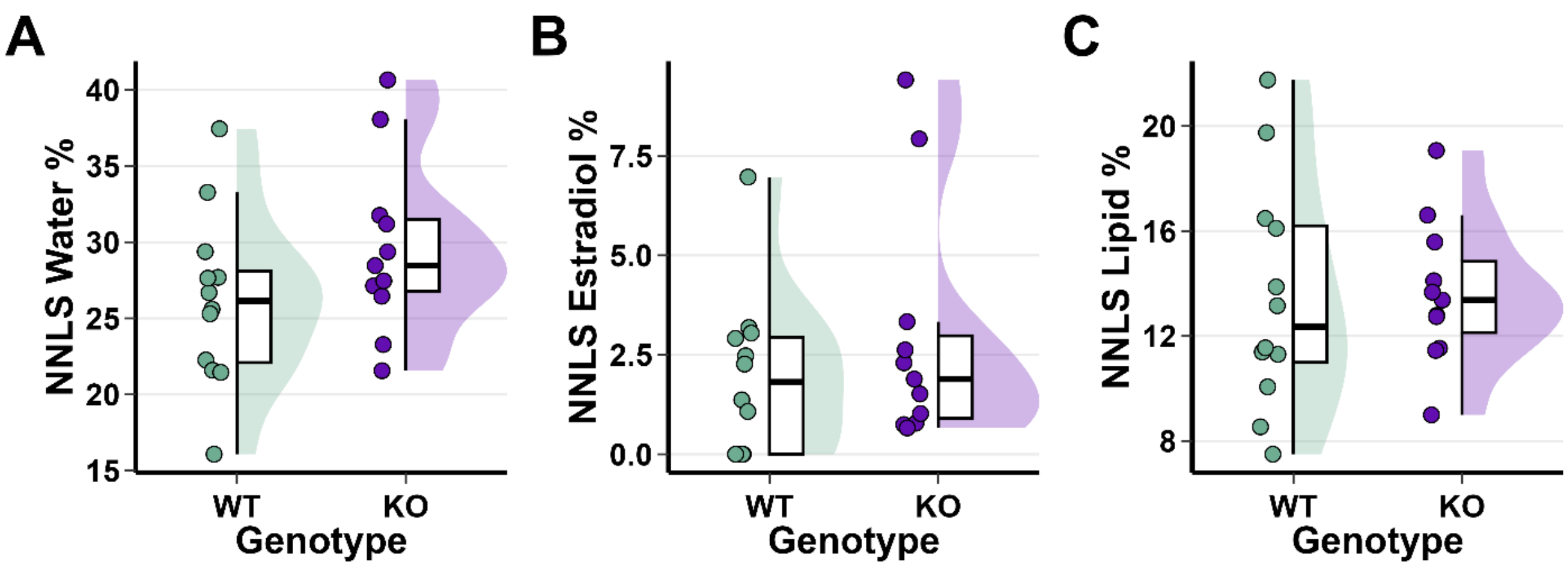

Figure S7. Water (p=0.19) (**A**), estradiol (p=0.48) (**B**), and lipid (p=0.65, phosphatidylcholine) (**C**) NNLS scores as percentages of the whole for wildtype (WT, MOPQ=0, green, n=12) and knockout (KO, MOPQ=2/3, purple, n=11) vagina. Each excised (5 x 5mm) ventral vaginal wall was subjected to sixteen RS measurements in grid format (Fig. 1A.3), and the results averaged for one data point per mouse. Boxplot data is represented as the mean +/- interquartile range. One knockout specimen was speckle coated prior to RS measurements, thus omitted from this section of analysis.

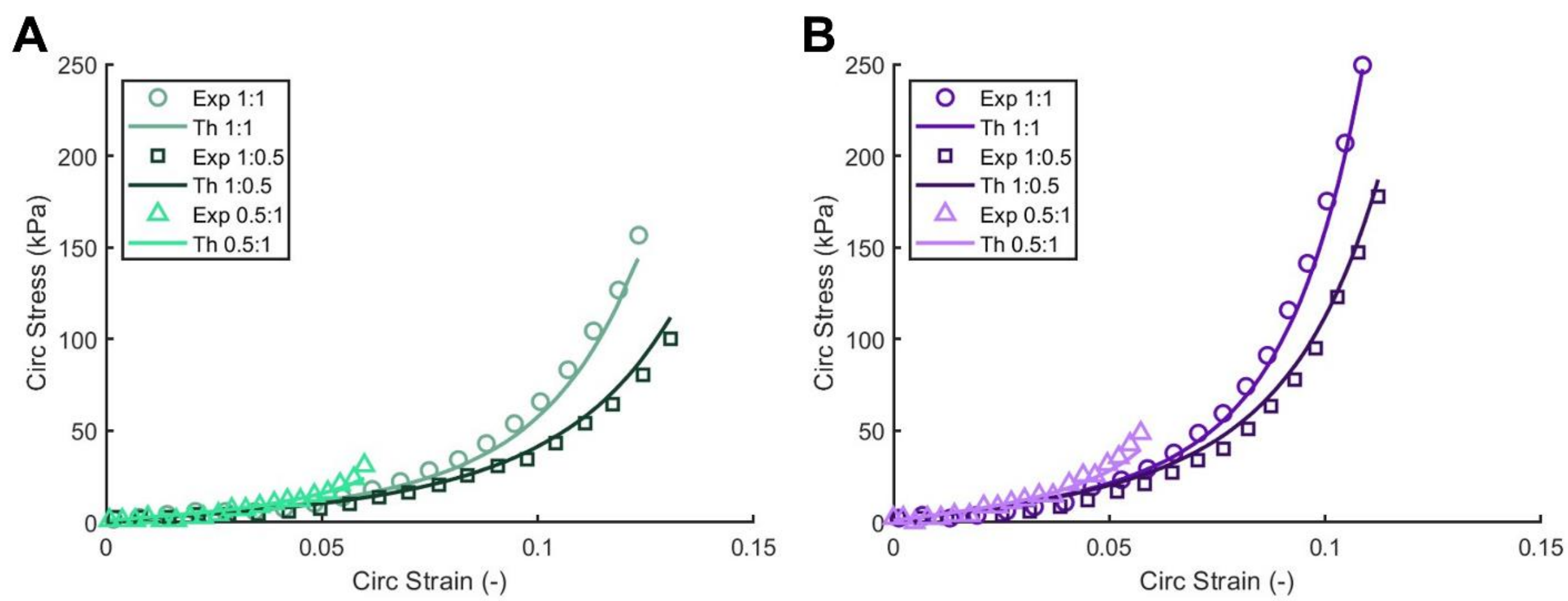

Figure S8. Representative Fung constitutive model fitting of stress-strain experimental data (scatter lines) and theoretical data (continuous lines) for three testing ratios including 1:1 (circles), 1:05 (squares) and 0.5:1 (triangles) displacement control ratios for both wildtype (WT, MOPQ=0, shades of green, **A**) and knockout (KO, MOPQ=2/3, shades of purple, **B**) vaginal tissue in the circumferential direction. Fung constitutive modeling results for each specimen are in Table S1.

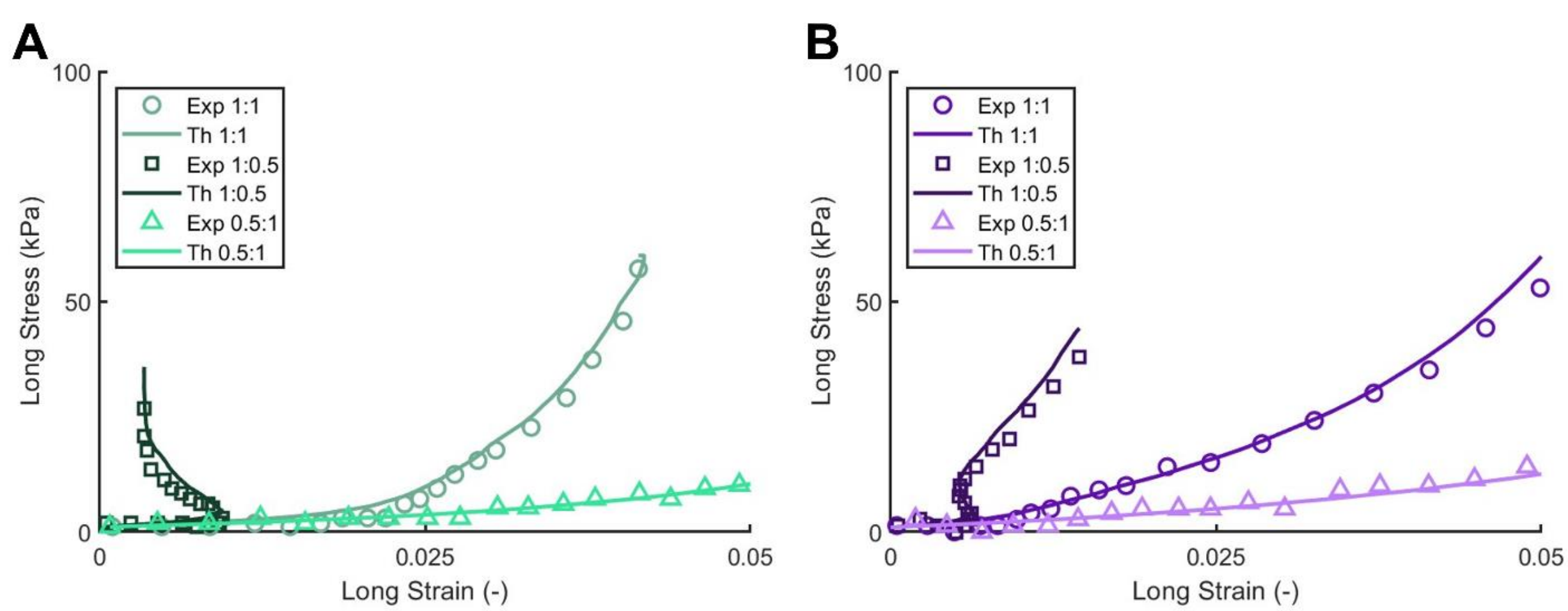

Figure S9. Representative Fung constitutive model fitting of stress-strain experimental data (scatter lines) and theoretical data (continuous lines) for three testing ratios including 1:1 (circles), 1:05 (squares) and 0.5:1 (triangles) displacement control ratios for both wildtype (WT, MOPQ=0, shades of green, **A**) and knockout (KO, MOPQ=2/3, shades of purple, **B**) vaginal tissue in the longitudinal direction. Fung constitutive modeling results for each specimen are in Table S1.

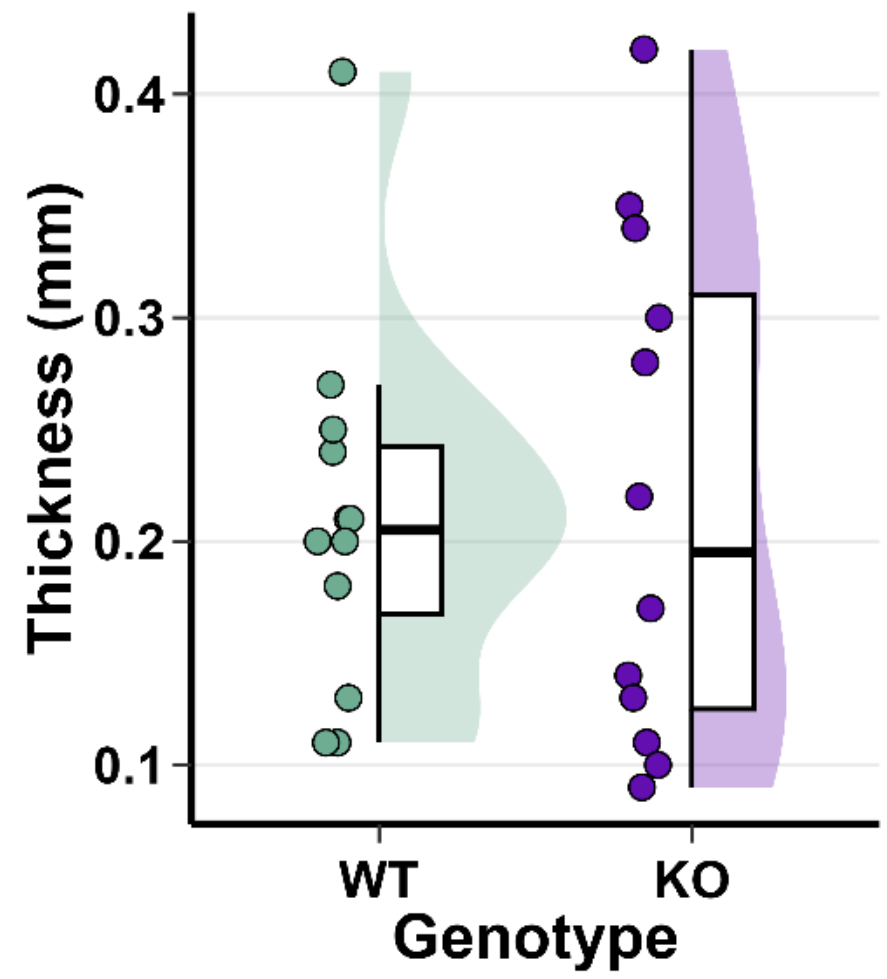

Figure S10. Total tissue thickness in millimeters acquired with a laser micrometer showed no significant differences (p=0.81) between the knockout (KO, MOPQ=2/3, purple) and wildtype (WT, MOPQ=0, green) vaginal samples (n=12/group) prior to testing. Boxplot data is represented as the mean +/- interquartile range.

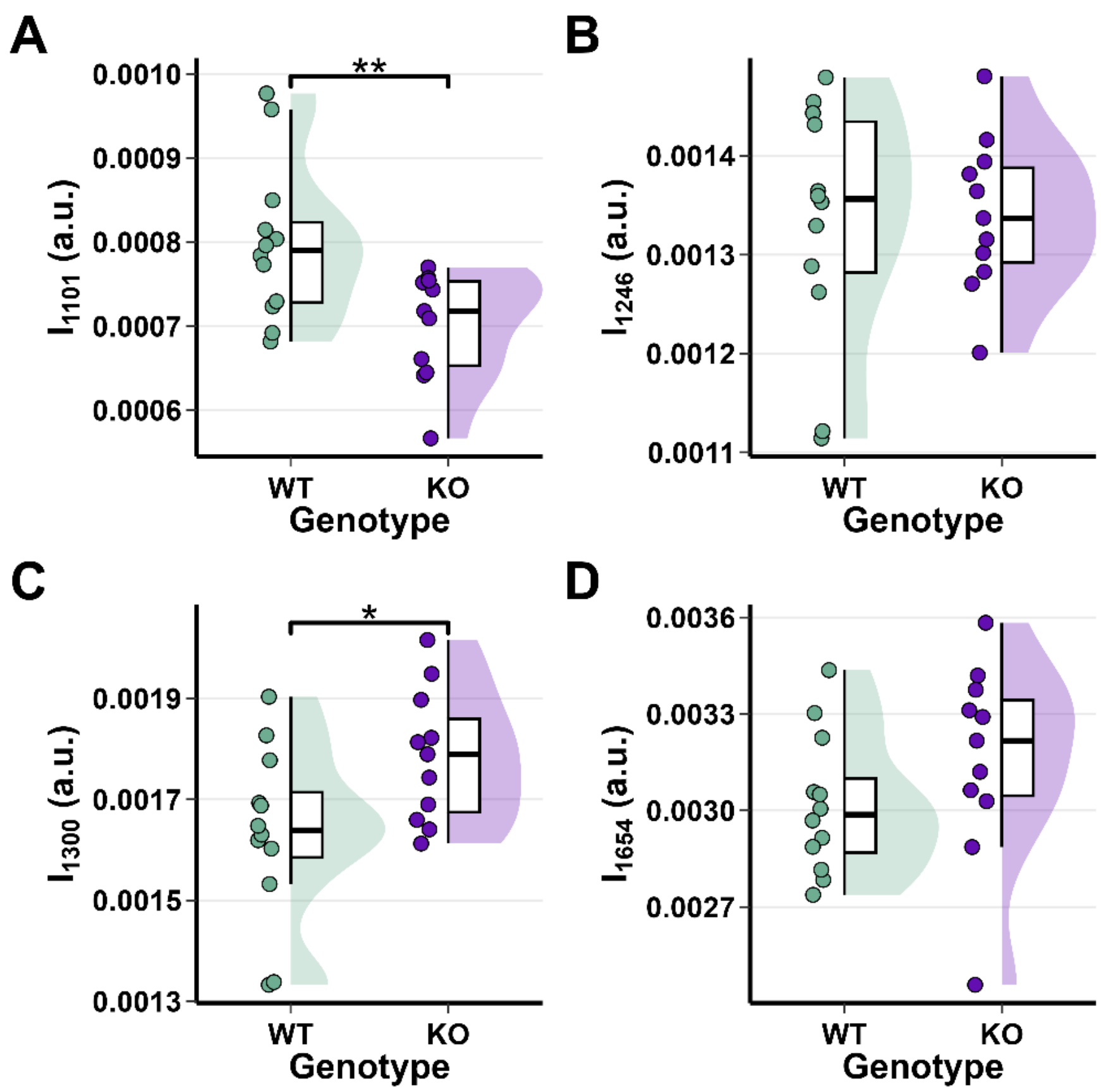

Figure S11. Raman peak intensity quantification to further investigate the defined peak ratios. Peak analysis identified significantly decreased I_{1101} (p=0.0086) (**A**) and significantly increased I_{1300} (p=0.044) (**C**); however, no changes were observed for I_{1246} (p=0.93) (**B**) and I_{1654} (p=0.12) (**D**) in the knockout (KO, MOPQ=2/3, purple, n=11) vagina compared to their wildtype (WT, MOPQ=0, green, n=12) littermates. Tentative peak assignments are found in Table 1. Each excised (5 x 5mm) ventral vaginal wall was subjected to sixteen RS measurements in grid format (Fig. 1A.3), and the results averaged for one data point per mouse. Boxplot data is represented as the mean +/- interquartile range. One knockout specimen was speckle coated prior to RS measurements, thus omitted from this section of analysis.

C. Supplemental Tables

Table S1. Fung constitutive model parameters and anisotropy index for murine vaginal tissues

Genotype	Model parameters						Anisotropy Index (φ)
	Sample ID	k (kPa)	c_1(-)	c_2(-)	c_3(-)	R^2	
Wildtype	FM001	1.21	103.14	46.75	35.37	0.98	0.59
	FM005	39.94	65.47	44.52	3.02	0.42	0.69
	FM006	3.38	53.62	3.64	1.82	0.98	0.10
	FM014	17.50	42.68	22.38	14.76	0.99	0.65
	FM015	4.32	47.78	16.56	8.40	0.98	0.44
	FM016	108.76	28.78	26.20	26.20	0.42	0.95
	FM017	1.89	223.56	82.14	65.91	0.99	0.51
	FM019	9.64	58.99	119.03	28.18	0.97	0.59
	FM020	4.19	145.46	59.33	44.78	0.99	0.55
	FM021	8.18	130.72	52.42	41.43	0.98	0.55
	FM027	2.53	117.83	17.29	17.29	0.97	0.26
	FM028	2.47	138.58	31.41	31.41	0.94	0.37
	**Mean**	**17.00**	**96.39**	**43.47**	**26.55**	**0.88**	**0.52**
	**SD**	**29.56**	**54.81**	**30.87**	**18.09**	**0.21**	**0.21**
Knockout	FM007	4.24	59.54	8.77	8.77	0.99	0.26
	FM008	2.38	130.10	12.71	10.39	0.98	0.16
	FM009	2.54	200.87	39.97	39.97	0.99	0.33

	FM010	8.82	85.84	23.98	11.81	0.97	0.37
	FM012	2.56	142.68	18.29	14.83	0.99	0.21
	FM013	6.57	38.11	9.96	1.52	0.96	0.29
	FM018	1.94	130.43	30.22	30.22	0.99	0.37
	FM022	0.61	218.21	24.19	24.19	0.94	0.20
	FM023	8.74	232.46	8.02	0.28	0.99	0.04
	FM024	27.55	64.49	55.23	22.81	0.99	0.89
	FM025	3.54	329.86	42.28	42.28	0.97	0.23
	FM026	7.37	167.40	31.50	31.50	0.99	0.32
	**Mean**	**6.40**	**150**	**25.43**	**19.88**	**0.98**	**0.31**
	**SD**	**6.90**	**81.48**	**14.37**	**13.54**	**0.01**	**0.20**

D. Supplemental References (as numbered in manuscript)

[41] M. S. Sacks, "Biaxial Mechanical Evaluation of Planar Biological Materials," *J Elast,* vol. 61, no. 1, pp. 199-246, 2000/07/01 2000, doi: 10.1023/A:1010917028671.

[45] E. K. Danso *et al.*, "Comparison of Biaxial Biomechanical Properties of Post-menopausal Human Prolapsed and Non-prolapsed Uterosacral Ligament," *Sci Rep,* vol. 10, no. 1, p. 7386, 2020/04/30 2020, doi: 10.1038/s41598-020-64192-0.

[55] E. E. Spurlin, K. Belcher, F. Shofu, J. A. E. Esteves, P. T. Jimenez, and C. M. O'Brien, "In vivo Raman spectroscopy reveals biochemical changes in the human cervix with pregnancy establishment," *npj Women's Health,* vol. 3, no. 1, p. 32, 2025/06/04 2025, doi: 10.1038/s44294-025-00083-x.

[56] C. M. O'Brien *et al.*, "In vivo Raman spectroscopy for biochemical monitoring of the human cervix throughout pregnancy," *Am J Obstet Gynecol,* vol. 218, no. 5, pp. 528.e1-528.e18, May 2018, doi: 10.1016/j.ajog.2018.01.030.

[57] C. M. O'Brien *et al.*, "In vivo Raman spectral analysis of impaired cervical remodeling in a mouse model of delayed parturition," *Sci Rep,* vol. 7, no. 1, p. 6835, Jul 28 2017, doi: 10.1038/s41598-017-07047-5.

[58] L. E. Masson *et al.*, "In vivo Raman spectroscopy monitors cervical change during labor," *Am J Obstet Gynecol,* vol. 227, no. 2, pp. 275 e1-275 e14, Aug 2022, doi: 10.1016/j.ajog.2022.02.019.

[62] M. J. E. Domingo, T. N. Vanoven, R. De Vita, M. E. F. Rodriguez, K. S. Miller, and I. J. Pence, "Biomechanical and Compositional Changes in the Murine Uterus with Age," *Ann Biomed Eng,* vol. 53, no. 6, pp. 1385-1398, Mar 24 2025, doi: 10.1007/s10439-025-03709-y.

[74] A. Rygula, K. Majzner, K. M. Marzec, A. Kaczor, M. Pilarczyk, and M. Baranska, "Raman spectroscopy of proteins: a review," *J Raman Spectrosc,* vol. 44, no. 8, pp. 1061-1076, 2013, doi: 10.1002/jrs.4335.

[87] M. Khalid, T. Bora, A. A. Ghaithi, S. Thukral, and J. Dutta, "Raman Spectroscopy detects changes in Bone Mineral Quality and Collagen Cross-linkage in Staphylococcus Infected Human Bone," *Sci Rep,* vol. 8, no. 1, p. 9417, Jun 20 2018, doi: 10.1038/s41598-018-27752-z.

[88] Y. C. Fung, K. Fronek, and P. Patitucci, "Pseudoelasticity of arteries and the choice of its mathematical expression," *Am J Physiol,* vol. 237, no. 5, pp. H620-31, Nov 1979, doi: 10.1152/ajpheart.1979.237.5.H620.

[89] C. Bellini and E. S. Di Martino, "A mechanical characterization of the porcine atria at the healthy stage and after ventricular tachypacing," *J Biomech Eng,* vol. 134, no. 2, p. 021008, Feb 2012, doi: 10.1115/1.4006026.

[97] D. E. Maidana *et al.*, "ThicknessTool: automated ImageJ retinal layer thickness and profile in digital images," *Sci Rep,* vol. 10, no. 1, p. 18459, Oct 28 2020, doi: 10.1038/s41598-020-75501-y.

[141] Y. C. Fung, "The Meaning of the Constitutive Equation," in *Biomechanics: Mechanical Properties of Living Tissues*. New York, NY: Springer New York, 1993, pp. 23-65.

[142] J. P. Vande Geest, M. S. Sacks, and D. A. Vorp, "The effects of aneurysm on the biaxial mechanical behavior of human abdominal aorta," *J Biomech,* vol. 39, no. 7, pp. 1324-34, 2006, doi: 10.1016/j.jbiomech.2005.03.003.

[143] S. Roccabianca, C. A. Figueroa, G. Tellides, and J. D. Humphrey, "Quantification of regional differences in aortic stiffness in the aging human," *J Mech Behav Biomed Mater,* vol. 29, pp. 618-34, Jan 2014, doi: 10.1016/j.jmbbm.2013.01.026.

[144] K. L. Billiar and M. S. Sacks, "Biaxial mechanical properties of the natural and glutaraldehyde treated aortic valve cusp--Part I: Experimental results," *J Biomech Eng,* vol. 122, no. 1, pp. 23-30, Feb 2000, doi: 10.1115/1.429624.

[145] Z. Movasaghi, S. Rehman, and I. U. Rehman, "Raman Spectroscopy of Biological Tissues," *Appl Spectrosc Rev,* vol. 42, no. 5, pp. 493-541, 2007/09/01 2007, doi: 10.1080/05704920701551530.
